# Perspectives and Impact of a Parent-Child Intervention on Dietary Intake and Physical Activity Behaviours, Parental Motivation, and Parental Body Composition: A Randomized Controlled Trial

**DOI:** 10.3390/ijerph17186822

**Published:** 2020-09-18

**Authors:** Shazya Karmali, Danielle S. Battram, Shauna M. Burke, Anita Cramp, Andrew M. Johnson, Tara Mantler, Don Morrow, Victor Ng, Erin S. Pearson, Robert J. Petrella, Patricia Tucker, Jennifer D. Irwin

**Affiliations:** 1 Health & Rehabilitation Sciences, Western University, London, ON N6A 3K7, Canada; skarmal@uwo.ca (S.K.); ajohnson@uwo.ca (A.M.J.); donmor@uwo.ca (D.M.); 2 Food and Nutritional Sciences, Western University, Brescia University College, London, ON N6G 1H2, Canada; dbattra@uwo.ca; 3 School of Health Studies, Western University, London, ON N6A 3K7, Canada; sburke9@uwo.ca (S.M.B.); tara.mantler@uwo.ca (T.M.); 4 Middlesex London Health Unit, London, ON N6A 3N7, Canada; anita.cramp@mlhu.on.ca; 5 Department of Programs and Practice Support, College of Family Physicians of Canada, Mississauga, ON L4W 5A4, Canada; vng@cfpc.ca; 6 School of Kinesiology, Faculty of Health and Behavioural Sciences, Lakehead University, Thunder Bay, ON P7B 5E1, Canada; erin.pearson@lakeheadu.ca; 7 Department of Family Practice, Faculty of Medicine, University of British Columbia, Vancouver, BC V6T 1Z3, Canada; robert.petrella@ubc.ca; 8 School of Occupational Therapy, Western University, London, ON N6A 3K7, Canada; ttucker2@uwo.ca

**Keywords:** overweight/obesity, parent-child dyad, coaching, physical activity, nutrition, motivation

## Abstract

Adults and children in Canada are not meeting physical activity guidelines nor consuming sufficient nutrient-rich foods. High engagement in these unhealthy behaviours can lead to obesity and its associated diseases. Parent-child interventions aimed at obesity prevention/treatment have assisted families with making positive changes to their nutrition and physical activity behaviours. Given that the home environment shapes early health behaviours, it is important to target both parents and children when addressing diet and physical activity. One method that has been shown to improve health outcomes is co-active coaching. The current study explored the impact of a three-month co-active coaching and/or health education intervention on the dietary intake and physical activity behaviours of parents with overweight/obesity and their children (ages 2.5–10; of any weight). Body composition (i.e., body mass index [BMI] and waist circumference), changes in parental motivation with respect to physical activity and dietary behaviours, and parental perceptions of program improvements were collected. A concurrent mixed methods study comprised of a randomized controlled trial and a descriptive qualitative design was utilized. Fifty parent-child dyads were recruited and randomly assigned to the control (*n* = 25) or intervention (*n* = 25) group. Assessments were completed at baseline, mid-intervention (six weeks), post-intervention (three months), and six-month follow-up. A linear mixed effects model was utilized for quantitative analysis. Inductive content analysis was used to extract themes from parent interviews. No significant results were observed over time for the dependent measures. Parents in both control and intervention groups reported varied program experiences, including developing changes in perspective, increased awareness of habits, and heightened accountability for making positive changes in themselves, and consequently, their families. Parents also shared barriers they faced when implementing changes (e.g., time, weather, stress). Qualitatively, both groups reported benefitting from this program, with the intervention group describing salient benefits from engaging in coaching. This research expands on the utility of coaching as a method for behaviour change, when compared to education only, in parents with overweight/obesity and their children.

## 1. Introduction 

If developed during childhood, obesity is likely to persist into adolescence and adulthood [1]. Moreover, children who have parents with overweight/obesity are at a high risk of developing the disease themselves, in that the family environment exerts an important influence on the development of children’s habits [2,3,4]. Given that it is more difficult to change health behaviours later in life, it is crucial to establish healthy habits from a young age [5]. 

Currently in Canada, 38% of 3–4-year-olds and 61% of 5–17-year-olds are not meeting recommended Canadian 24-h Movement Guidelines for their age groups (>60-min of moderate-to-vigorous intensity physical activity (MVPA per day [6,7]). In addition, sedentary behaviours are on the rise, with 76% of 3–4-year-olds and 51% of 5–17-year-olds engaging in more screen-viewing time than is recommended in the Canadian 24-h Movement Guidelines for recreational screen-based sedentary behaviours [8]. These age groups spend 2.3 h (5–11 year-olds) and 4.1 h (12- to 17-year-olds) engaged in recreational sedentary behaviours (e.g., watching television, text messaging, video games) per day [8]. The increased time engaged in sedentary behaviours is concerning, in that it takes away from time that could be spent being physically active, and increased sedentary behaviour poses a health risk independent of MVPA levels [8,9]. Regular engagement in physical activity (PA) helps to reduce depressive symptoms and anxiety in children and also enhances their stress responses, resiliency, self-esteem, self-concept, and self-perception [8,9]. In turn, positive functioning in these areas can promote better moods, increase life satisfaction, and minimize negative impacts of stress [10,11,12]. Participating in PA, such as sports, helps to develop positive physical, psychological, and social functioning [10,12]. Children who play sports are more likely to continue engaging in PA into adulthood [13]. Unfortunately, significant declines in sport participation have been reported as children transition into adolescence, with a sharper decline noted in females’ participation rates than males’ at this life stage [13]. If a female has not participated in a sport (organized or individual) by the age of 10, there is only a 10% chance that she will be physically active as an adult [13]. 

Children whose parents are active are more likely to be active themselves [14]. Surprisingly, only 38% of Canadian parents with 5–17-year-olds report playing active games with them [13]. In addition, parents’ own PA engagement is very important. As noted by Garriguet, Colley, and Bushnik [15], who studied a sample of over 1300 parent-child pairs, every 20-min increase in parental MVPA was associated with 5–10-min increases in the MVPA of their 6–11-year-old children, independent of parental support for PA. Interestingly, only 16% of Canadian adults meet the current guidelines of 150 min of MVPA per week [16]. 

In addition to their deficits in PA levels, one in five Canadian children (ages 1–8) have energy intakes that exceed their energy needs [17]. Inadequate nutrition in children can lead not only to the development of obesity, and its associated diseases, but also can impact brain development, leading to psychosocial and behavioural problems [18,19,20]. Among Canadian adults aged 19 and over, 50% of women and 70% of men have energy intakes that exceed their needs [21]. Foods that are high in sodium, free sugars (i.e., added sugars), and saturated fat—deemed nutrients of concern—contribute to an increased risk of chronic diseases when consumed in excess [22]. Examples of these foods include cheese, red meat, sugar-sweetened beverages, and pre-packaged meals [22]. In 2017, 58% of all Canadians consumed sodium above the recommended limits, and one in two Canadians consumed higher than recommended levels of saturated fat [22]. 

As previously outlined, parents and the home environment are key influences on the development of children’s health habits [2,4]. Parents/caregivers can affect children’s eating behaviours by making nutritious food choices for the family, modeling dietary choices and patterns, and using feeding practices to reinforce the development of eating patterns and behaviours [2,4]. Eating behaviour is taught through parental examples; for instance, children’s intake of fruits, vegetables, and milk often increases after observing adults consuming those foods [23,24]. Food parenting practices are strategies parents use to influence the amount and types of foods their children eat [24,25]. Role modeling healthy eating behaviours, involving children in food decisions, and encouraging a balanced and varied diet are feeding practices parents must be made aware of, as those habits have been associated with healthy diets and body mass indices (BMI) in children [25]. Given that parents play a formative role in shaping their children’s health behaviours, it is imperative to provide parents with effective resources and supports aimed at raising awareness and increasing knowledge to enhance healthy behaviours in themselves and their families [25]. 

Parental motivation to engage in program tasks has been a large determinant of the success of health promotion interventions for parents and their children [3]. Motivation has been conceptualized as an individual’s readiness to change a behaviour—measured as the degree to which the person feels change is important—and their level of confidence in their ability to implement that change [26,27]. In programs targeted at improving obesity-promoting behaviours in children, parental motivation has been significantly associated with: (a) the promotion of healthy behaviours (i.e., dietary and PA changes) in their children, (b) a reduction in child BMI-z, and (c) the successful completion of program tasks by parents [28,29,30,31]. When compared to those with high levels of motivation, parents with low motivation at baseline of family-based behavioural treatment for childhood obesity were less likely to complete the full program [28,32]. Goal-setting and reinforcement intervention strategies have been found to increase parental motivation to change PA behaviour, even in the face of constraints such as time or scheduling difficulties; goal setting may provide busy parents with the additional incentive needed to prioritize their child’s PA over other tasks [33]. Therefore, addressing and facilitating parental motivation to engage in healthy behaviours is important for their and their child’s success in obesity prevention and treatment interventions [28]. 

In a systematic review of family-based lifestyle interventions for weight loss and weight control in children and adolescents (ages 2–19), Sung-Chan, Sung, Zhao, and Brownson [34] reported that these interventions produced positive effects regarding weight loss in children with overweight/obesity, and that family played an important role in modifying the nutrition and PA behaviours of these children. This finding was supported by ref. [33] in their review of family-based interventions to increase PA in children, in that increases in PA behaviours by one family member prompted others in the same family to engage in activity themselves. Similarly, in an intervention in which parents were asked to track their daily activity using pedometers, and also received weekly telephone calls focused on reflection and encouragement for behaviour change, reported appreciating the reinforcement they received from these weekly calls [32]. The authors hypothesized encouragement received during telephone calls motivated parents to change their children’s PA behaviours, as well as their own. The researchers of the aforementioned review [33] also found that while providing parents with health education was an effective intervention for changing PA knowledge, health education supplemented with reinforcement was more successful in producing behaviour change [33]. 

One method that has potency in eliciting positive improvements in behaviour change in various areas (e.g., smoking cessation; obesity treatment; mental health; [35,36,37,38]) is Co-Active Life Coaching (CALC; [39]). This primarily telephone-based method is a specific style of life coaching that centers on viewing clients as the expert in their own lives. The coach’s role is to assist the client in deepening their understanding of themselves and/or moving toward meaningful actions of their choosing [39]. This method of coaching allows clients and coaches to work collaboratively, not necessarily to focus on specific behaviours, but on any area of the client’s interest, which often result in behaviour changes. Previous research studies utilizing CALC as an intervention for obesity among adults have demonstrated significant improvement in physical and psychological outcomes [37,38]. 

### Study Purpose

The purpose of this study was to identify the impact of a three-month parent-focused coaching plus health education intervention compared to three-month parent-focused health education only on: (a) the PA levels and dietary intake of children (ages 2.5–10) and their parents with overweight/obesity, (b) parental motivation to engage in healthy behaviours, and (c) parental body composition (i.e., BMI and waist circumference). Parents’ perspectives of how the intervention(s) influenced their own, their children’s, and their family unit’s nutrition and PA behaviours were explored, in addition to their perceptions of what would have made the program more effective for behaviour change. 

## 2. Materials and Methods

### 2.1. Design

**Trial registration:** ISRCTN ISRCTN69091372. Retrospectively registered 24 September 2018.

A concurrent mixed methods study comprised of a randomized controlled trial and a descriptive qualitative design was utilized as the analytical framework for this study. Utilizing this type of mixed methods research design, in which both qualitative and quantitative data are collected, allows for both types of data to complement and supplement each other [40]. This permitted the researchers to gain a more complete understanding of participants’ experiences, which could not have been obtained by employing only one approach [40]. 

Parent-child dyads were randomly assigned to either: coaching plus health education (intervention) or health education alone (control). Ethical approval was received from the host institution’s Health Sciences’ Research Ethics Board (ID# 109219). The methods pertaining to the protocol and a complete intervention description have been published elsewhere [41]. However, as the current study progressed some necessary adjustments were made to the protocol. Thus, a brief procedural account is described below. 

#### 2.1.1. Participants and Recruitment

Parents were recruited through flyers, Facebook and Twitter posts, Kijiji advertising service site/s, a local radio advertisement, and advertisements in neighborhood and parent-focused magazines. To be eligible for this study, parents/guardians were required to have a BMI of ≥25 kg/m^2^, live with their child (ages 2.5–10) for at least 5 days/week, speak English, and be comfortable using a computer for data collection. Recruitment and enrollment occurred from August 2017-November 2018. Once a parent-child dyad was determined as eligible to participate, a baseline appointment was made to obtain informed consent, conduct parent measurements, inform the parent of group assignment, and provide the dyad with pedometers. All dyads consisted of one parent and one child; the participating parent received the intervention and/or health education. 

The current study employed an active control group, in which participants received health education, as opposed to utilizing a no treatment control. This decision was made on ethical grounds [42], in order to provide parents who wanted to make changes in their and their children’s behaviours with resources that may help them do so. Researchers have reported that health education alone is an effective method to enhance knowledge toward behaviour change [43,44]. Moreover, from a health promotion perspective, providing individuals with knowledge to make healthy choices would allow them to increase control over their own health [45]. Therefore, health education was selected as an appropriate active control condition for this research. 

#### 2.1.2. Health Education Modules (Webinars)

Parents in both the control and intervention groups received six evidence-informed (e.g., [6,46,47] online health education (designed to be approximately 20–30 min each) sessions (or webinars) in module format. Three sessions focused on PA and lifestyle behaviours (i.e., benefits of PA, guidelines, sedentary behaviour, sleep, physical literacy, ideas for increasing PA in daily lives of parent and child, and local resources to help increase PA), and three sessions focused on nutrition (i.e., understanding nutrients and nutrition labels, eating with children, positive food environments, barriers to healthy eating, and healthy eating on a budget). Once participants joined the study, the modules were made available on the host university’s eLearning platform. Parents were asked to engage in their next lesson approximately 7–10 days after their previous one.

#### 2.1.3. CALC Plus Health Education Intervention

Parents in this intervention group received CALC in addition to the online health education modules. Participants received nine, 20–30 min, one-on-one, telephone-based coaching sessions (three/month for 3 months) focusing on the agenda of the parent’s choosing. The lead researcher randomly paired parent participants with a Certified Professional Co-Active Coach (CPCC) and together they scheduled their sessions. The parent determined the agenda for each session, and the coach was asked to use only their CPCC skills (e.g., asking genuinely curious open-ended questions, reflecting back what the participant says, acknowledging the participant’s experience, and championing their progress; [39]).

#### 2.1.4. Certified Professional Co-Active Coaches

The CPCC training program is intensive, and requires individuals to first complete two courses on CALC, followed by a 6-month certification program (which runs 3–5 h every week) [48]. In addition, trainees must work with a CPCC for one hour per month. The Co-Active coaching and certification program is accredited by the International Coach Federation (ICF) [48]. Sixteen CPCCs were invited to deliver the intervention; of those invited, 12 agreed to coach in this study. Upon participant enrollment, coaches were assigned one to three parents—based on how many parents each coach identified as suitable for them. Coaches received an honorarium.

### 2.2. Data Collection

Rolling recruitment was employed, tailoring the data collection periods to each participant. Data were collected at baseline (i.e., 1 week prior to the start of the intervention); mid-intervention (i.e., 6-weeks into the intervention); immediately post-intervention (i.e., 3 months after the intervention began); and finally, at 6 months post-intervention. Parent participants were asked to complete demographic information forms on behalf of themselves and their child at baseline. Both groups completed the same assessments at each follow-up period. The lead researcher and a research assistant conducted baseline and follow-up assessments at either the host university or at the participant’s home. One week prior to follow-up times, an email link was sent to parent participants asking them to complete the questionnaires, and email, text message, and telephone reminders were sent 1 and 2 weeks later if no response was received. If a participant could not be contacted after three consecutive communication attempts, they were contacted again at their next follow-up time, and if no response was received at that point, it was assumed that they were withdrawing from the study. A grocery store gift card was provided to dyads who completed the study.

### 2.3. Measures

#### 2.3.1. Pedometer and 24-h Food Recall (Parent and Child)

At each assessment point, parents and children were asked to wear a pedometer for one week (including weekdays and weekends). In addition, parents were asked to track their and their child’s food intake for a full 24-h, in as much detail as possible (e.g., portion sizes, brand). To calculate parent and child nutrient consumption at each time-point, dietary recall records were entered into a food processing computer program (The Food Processor, Nutrition and Fitness Software, ESHA Research, Version 11.3.285, Oregon, United States of America). Trained nutrition students reviewed all food records to ensure accuracy of foods and portion sizes. After consulting with qualified dieticians at the host university, the following nutrient intakes were chosen for analysis in parents and children: protein, fiber, saturated fat, and sodium. Total caloric intake was analyzed in parents only, as it was suggested that this is not an impactful measure in child populations.

#### 2.3.2. Height, Weight, and Waist Circumference (Parent)

The lead researcher and a research assistant conducted parental anthropometric measurements at each assessment time. This consisted of measuring height and weight (to calculate BMI), and waist circumference. Weight was measured using a SECA 803 digital floor scale (SECA, Chino, CA, USA), and height was measured using the SECA 207 mechanical stadiometer (SECA, Chino, CA, USA; at the host institution), or the SECA 217 portable stadiometer (SECA, Chino, CA, USA; for at-home follow-ups). Waist circumference measurements were obtained following Heart and Stroke Foundation [49] guidelines, whereby the measuring tape is placed at the midpoint between the bottom of the ribcage and the iliac crest along the ancillary line.

#### 2.3.3. Standardized and Validated Questionnaires (Parent)

Parents were asked to complete standardized and validated questionnaires at each assessment point, which were available on the online survey software Qualtrics (Provo, Utah, USA). The International Physical Activity Questionnaire (IPAQ; [50]) is a self-report measure of time spent performing physical activity (in metabolic equivalent (MET) minutes) and time spent sitting during the week. The IPAQ short-form was used in this research. Outlier and truncation instructions were followed. The Treatment Self-Regulation Questionnaire (TSRQ; [51]) is utilized to assess different forms of motivation as they relate to engaging in PA and a healthy diet. It is comprised of three subscales: (a) amotivation (i.e., absence of motivation; no meaningful relation between what they are doing and themselves); controlled motivation (i.e., behaviour that is performed to obtain a reward or to avoid negative consequences; behaviour performed to avoid feeling guilty; internally controlled but not self-determined); and (c) autonomous motivation (behaviour that is positively valued by the individual; behaviour is perceived as being part of the larger self and connected to other values and behaviours that may or may not be health related; self-determination that underlies behaviours that are engaged in for interest and pleasure from performing them). The TSRQ is measured on a Likert scale of 1 (not at all true) to 7 (very true), with higher scores indicating higher motivation [52].

#### 2.3.4. In-Person Interviews (Parent)

To best understand changes in experiences through the duration of this program, semi-structured, one-on-one interviews were conducted with parents at each follow-up assessment. Open-ended questions for the qualitative interviews (Table 1) were derived based on previous coaching studies [53,54], as well as the researchers’ expertise, to garner parents’ perceptions of how the interventions impacted them, their child, and their families, as well as program aspects that would have been more effective in eliciting behaviour change.

Baseline interviews were conducted to understand motivations for joining this study, and because these data fall outside the purpose of this paper, the findings will be presented elsewhere. Qualitative findings included in this paper outline parents’ perceptions of the impact of the program over time on the aforementioned outcomes, and program improvements that would have been more effective for behavior change. Thus, themes from mid-, post-, and 6-months post intervention are presented. The lead researcher conducted the audio-recorded interviews that were transcribed verbatim, and a research assistant also took notes.

### 2.4. Analysis

Due to high participant drop out, as presented below, and some participants not completing questionnaires at each time point, there was substantial missing data in all data sets. For these reasons, a mixed effects model was considered as the analytic method of choice, as this allows for the use of all available data [55].

A linear mixed effects model was utilized, with group (intervention versus control), and time (baseline, mid-intervention, post-intervention, and 6 month follow-up) entered as fixed effects to explore the impact of the coaching intervention (as compared to education only) on: parent and child step counts and dietary intake; parental BMI and waist circumference; and IPAQ and TSRQ scores. Each of the dependent variables were evaluated within separate models. Per-comparison alpha was adjusted for multiple comparison bias (i.e., when comparing the results to an alpha of 0.01). One participant was a significant outlier, in terms of caloric intake, throughout the study and was removed from the sample prior to data analysis. All statistical analyses were performed using R version 3.6.1 [56], with linear mixed effects analyses conducted using the lme4 [57] and lmerTest [58] packages. Post-hoc comparisons amongst the time periods were assessed using the emmeans package [59].

Interviews were transcribed verbatim, and two researchers independently completed inductive content analysis to identify common themes [60]. Strategies employed to uphold data trustworthiness are presented elsewhere [41]. Due to the differences in ‘treatment’ received by control and intervention groups, interviews were analyzed separately at each follow-up time point. Although quotations may be relevant to more than one theme, they are presented in the section in which the quote best fits.

## 3. Results

**Figure 1:**
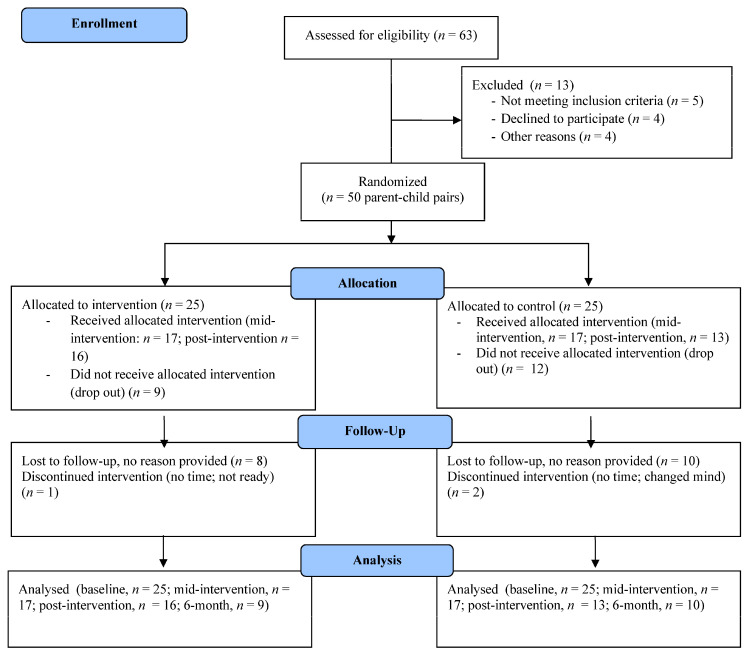
CONSORT diagram showing retention and attrition in current study.

In total, 50 parent-child dyads enrolled in this study; the majority of parents and children were female and the average age was 37 (6.7) years and 6.8 years (2.8), respectively. Demographics for these individuals can be found in Table 2. Due to attrition, all participants did not complete assessments at every follow up time (i.e., six-week, *n* = 34; post-intervention, *n* = 29; six-month, *n* = 19). Site statistics showed that 68% (*n* = 17) of intervention group parents and 80% (*n* = 20) of control group parents accessed the health education webinars. For a full outline of retention and attrition, see CONSORT diagram (Figure 1).

### 3.1. Quantitative Results

Given that there were only three males, we opted to control for sex effects through the application of homogeneous subset selection (i.e., only female participants were selected for quantitative analysis). Thus, these results should only be generalized to female parents. No participants were deleted from the child data set.

#### 3.1.1. Child PA and Dietary Intake

Results from children’s PA and dietary intake are presented in Table 3. 

These included steps per week, protein intake, fiber intake, saturated fat intake, and sodium intake. The main effects model demonstrated no significant difference from the null model over time on: children’s step count, children’s protein intake, children’s fiber intake, children’s saturated fat intake, or children’s sodium intake. The interaction model also demonstrated no significant difference from the null model, suggesting that there was also no effect of the intervention over time on the aforementioned outcomes.

#### 3.1.2. Parent PA, Dietary Intake, and Anthropometric Variables

Parental PA outcomes from each follow-up point are presented in Table 4, including step count (over the course of one week), BMI, waist circumference, IPAQ MET minutes per week, and sitting minutes per day.

The main effects model demonstrated no significant difference from the null model on parental: BMI, waist circumference, steps per week, IPAQ MET minutes, or IPAQ sitting minutes per day. The interaction model demonstrated no significant difference from the null model, suggesting that there was no effect of the intervention over time on parental: BMI, waist circumference, number of steps per week, or IPAQ sitting minutes per day.

Parental nutritional outcomes from all follow-up points are presented in Table 5, including caloric intake, protein, fiber, saturated fat, and sodium.

The main effects model demonstrated no significant difference from the null model when adjusting for multiple comparison bias (i.e., when comparing the results to an alpha of 0.01) on parental: caloric intake, protein intake, fibre intake, or saturated fat intake. The interaction model also demonstrated no significant difference from the null model on the aforementioned outcomes.

**Figure 2:**
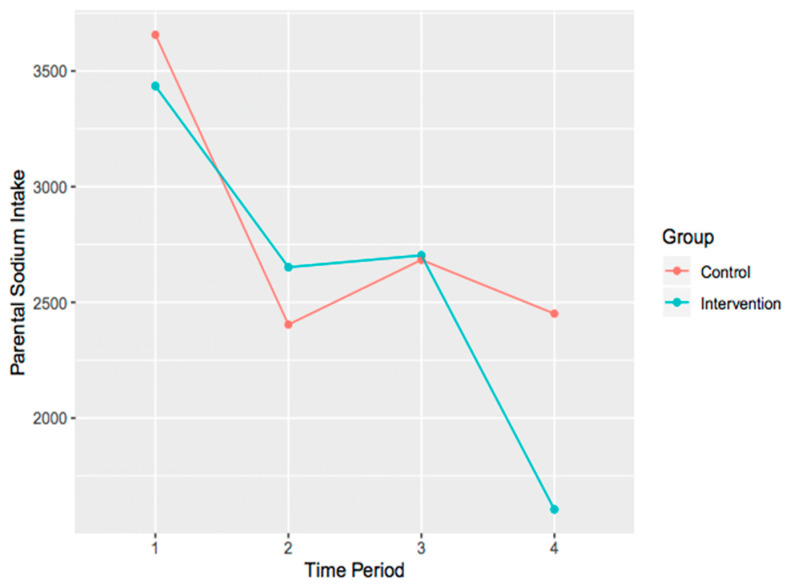
Interaction plot of parental changes in sodium intake over time for both control and intervention groups.

The main effects model demonstrated no significant difference from the null model, χ^2^ (4) = 13.25, *p* = 0.01, when adjusting for multiple comparison bias (i.e., when comparing the results to an alpha of 0.01) on parental sodium intake. Similarly, the interaction model demonstrated no significant difference from the null model, χ^2^ (7) = 15.09, *p* = 0.04, when adjusting for multiple comparison bias. It is important to note however, the trend that is present within the data (i.e., the effect ‘approached significance’). To explore this, interaction plots were created (Figure 2).

#### 3.1.3. Parental Motivation

Parental motivation was assessed using the TSRQ. Outcomes from each follow up time, for diet and exercise, are presented in Table 6. These outcomes included autonomous motivation, controlled motivation, and amotivation.

The main effects model demonstrated no significant difference from the null model on: diet autonomous motivation, diet controlled motivation, diet amotivation, exercise autonomous motivation, exercise controlled motivation, or exercise amotivation. The interaction model also demonstrated no significant difference from the null model, suggesting that there is no effect of the intervention over time on the aforementioned outcomes.

### 3.2. Qualitative Findings

**Figure 3:**
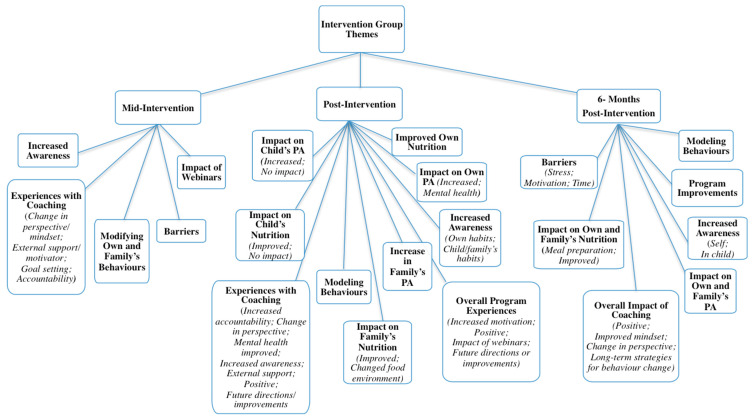
Intervention themes from all relevant time points.

**Figure 4:**
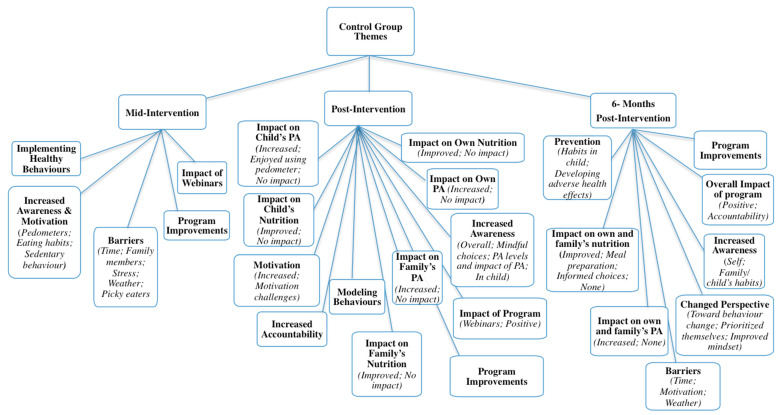
Control themes from all relevant time points.

Qualitative interviews with parents resulted in a vast number of supporting statements (N_words_ = 13,028, approximately), far more than could be included in the current manuscript. Duration of interviews varied at each follow-up time: 5–41 min (mid); 5–57 min (post); and 6–39 min (six-months). Data saturation was reached for each time point. To avoid repetition, themes and/or subthemes that were unique to each follow-up point are presented and described. For a complete outline of themes, organized by group and follow-up time, see Figure 3 and Figure 4.

#### 3.2.1. Mid-Intervention Themes

Intervention group themes. Five themes and four sub-themes were identified from mid-intervention follow-up interviews with intervention group participants (*n* = 17) with regard to impact of, and experiences in, the program. Corroborative quotations for each theme can be found in Table 7.

Experiences with coaching: Most participants in the intervention group expressed that, at the six-week mark, coaching had positively impacted their lives. They described developing changes in their perspectives toward behaviour change; the benefit of having an external supporter or motivator; improvements in goal setting skills; and experiencing increased accountability to themselves and their coaches. For a full explanation of participants’ experiences with coaching, as well as coaches’ perspectives, and corroborative quotations please see ref. [61].

Increased awareness: Parents described an increase in awareness of their and their child’s habits, as well as their reasons for engaging in unhealthy behaviours. Many participants expressed that they turned to unhealthy foods when they were stressed or when they were having a bad day, and some mentioned that the foundation of their unhealthy nutrition habits stemmed from experiences in their childhood. They became more aware of the importance of engaging in self-care, and how focusing on their mindset led to improvements in other areas of their lives. Overall, participants expressed that though coaching sessions did not necessarily centre on nutrition and PA, they realized that improving their mental health resulted in improving their habits and behaviours. In addition, participants became more aware of their and their children’s PA habits through the pedometers. Some parents realized that their children were gaining higher step counts during the week when they were at school or daycare and fewer on the weekends. In addition, they became more aware of their own number of steps taken per day, as well as their levels of sedentary behaviour.

Modifying parental and family behaviours: Participants spoke about changes they made for themselves, and within their households. This included increasing the amount of fruits and vegetables they gave their children, as well as altering the types of foods and snacks they consumed themselves. In addition, participants started to increase their own engagement in PA, via walking, yoga, circuit training, skipping, and going to the gym. In some cases, these activities were done with their children.

Impact of webinars: Parents in the intervention group explained the impact of the educational webinars on their behaviours. Many said that they felt they already knew most of the information that was provided. Others said that they learned new information regarding nutrition labels, percent daily values, physical activities for their children, and about how food should not be used as a reward.

Barriers to behaviour change: Those parents who spoke about barriers explained that barriers prevented them from changing their current behaviours; these included weather, lack of time, and cost. They also felt they didn’t have enough time to exercise, that weather prevented them from being active outdoors, and gym memberships and healthy foods were too expensive.

Control group themes: Five themes and eight subthemes emerged at mid-intervention from interviews with parents in the control group (*n* = 17) regarding impact of, and experiences in, the program. Corroborative quotations for each theme can be found in Table 8.

Increased awareness and motivation: Parents in the control group explained that the pedometers made them aware of how much daily activity they and their children were acquiring. Many said that their children thoroughly enjoyed tracking steps, particularly because they would compete with their participating parent. Children, according to some parents, felt motivated to gain more steps when they realized that their step count numbers were higher than their parents’. Parents also began to take notice of the amount of screen time in which their children were engaging. Similar to the intervention group, parents in the control group expressed that they were surprised to learn how few steps they acquired on certain days and were also more aware of how much time they spent in sedentary behaviours. It was also shared that the dangers of prolonged bouts of sitting came as a shock to some participants. In addition to heightened awareness of PA habits, participants started to make changes to their and their family’s nutrition. They noticed the types of foods their children were consuming. Some parents explained that their motivation to change stemmed from realizing that their unhealthy habits not only affected themselves, but their families as well. In addition, the impact of modelling behaviours was also realized.

Implementing healthier choices: Parents described changes they started to make for themselves, and in their households. Some explained that they had resisted the temptation to buy fast foods, others said they began introducing more fruits and vegetables into their homes, and some described that they were switching unhealthy food choices for healthier ones (e.g., carbonated water instead of soda pop). Many parents also said that they had removed unhealthy treats from their homes and had decreased sugary snacks from their children’s diets. Some parents noticed the positive impact healthy eating was having on their and their child’s mindset in that they perceived their children as feeling better, having improved concentration, higher energy, and presenting with less agitation. Participants expressed that they had been making a conscious effort to increase their daily PA by bike riding, increasing their walking through parking further away from destinations, or taking the stairs more frequently. Some parents shared the information from the education sessions with their partners and/or families. They began involving their children in cooking and increasing PA with their families (e.g., going for walks together).

Impact of webinars: Similar to those in the intervention group, members of the control group reported that the education sessions were a refresher of information they mostly already knew. They explained that though they knew the information, they found it helpful and appreciated having reminders of what healthy habits entail regarding PA and nutrition. Some parents expressed that they learned new information from the webinars such as reading nutrition labels and differences in serving sizes.

Barriers to behaviour change: Some participants in the control group described that because there was no accountability piece for them and the webinars were self-led, they forgot to continue accessing them. A few participants explained that they had difficulty finding time to complete the webinars because they felt daily life was too hectic. Others spoke about barriers including picky eaters in their families and unsupportive partners who did not want to change their dietary habits. In addition, some parents noted that the weather prevented them from being more active, and that other family members made unhealthy foods too available to their children (e.g., grandparents).

Program improvements: Parents in the control group explained that, while they found the webinar information helpful, they wanted more frequent check-ins with the researchers to serve as support to keep them on track and accountable. A few parents stated that they wanted a more structured program to help them complete the webinars, as they found the self-led format challenging. The desire for assistance with addressing mental health challenges was also expressed.

#### 3.2.2. Post-Intervention Follow-Up Themes

Intervention group themes: Ten themes and 21 sub-themes were identified from immediate post-intervention interviews with intervention group participants (*n* = 16) with regard to the impact of the program on themselves and their families. Eight of these themes and 12 sub-themes were unique to this follow-up time, and therefore will be described in detail below. Corroborative quotations for each new theme/sub-theme are presented in Table 9.

Impact on child’s nutrition: Most parents explained that they felt their children’s dietary intake had improved over the course of the program. Due to meal planning and making healthier foods more available in their homes, their children were making better nutritional choices than before the program. Parents reflected that their children’s preferences seemed to change in that instead of craving convenience or fast foods, they were more likely to select a fruit or vegetable when they wanted a snack. They explained that, before the intervention, their children enjoyed and craved fast foods and sugary snacks. Conversely, some parents felt that their children already had well-balanced diets. Parents explained that they tried to ensure their child’s habits were healthier than their own and had always made healthy food available in their homes. These parents they felt the program did not impact their child’s dietary intake.

Impact on child’s PA: Intervention group parents’ perceptions of their children’s PA levels varied; some felt levels increased while others felt there was no change. Some parents reported that children who observed their parents becoming more active began to increase their own activity as well. Parents explained that because they felt accountable for their child’s PA levels, they encouraged their child to be active, and became more cognizant of their child’s activity levels. Through increasing their daily PA, children engaged in less sedentary behaviour (such as watching television) and spent more time playing outside. A few parents noted that this was a vast change compared to before the program in that their children tended to be sedentary after school and/or their extra-curricular activities. Parents also noted that their children enjoyed using the pedometers; they sensed that using pedometers encouraged higher step counts in their children. Parents felt that, from this increase in PA, their children were also motivated to join other sporting activities. Some parents felt that because their child was already active, and remained so throughout the program, their PA levels did not change. Parents opined that because their children were very active during the days, whether through daily PA or structured activities, increases in their PA due to the program were unlikely.

Improved own nutrition habits: All parents in the intervention group felt that the program made at least some impact on their dietary intake and helped them improve their nutrition behaviours. They explained that instead of choosing quick and convenient foods, as they would have done before the program, they were more likely to choose healthier foods. They became cognizant about the choices they made and implemented what they felt were manageable changes. Parents explained that meal planning and preparation helped them with making the healthier choice more convenient in that these foods were now readily available in their homes. Moreover, in making small changes in their diets, participants found their food preferences changed over the course of the program (e.g., craving less sugar).

Impact on own PA: Some parents in the intervention group noted substantial improvements in their PA levels through their involvement in the program. Participants shared that they incorporated PA into their days such that PA became a part of their routine. For instance, they would take stretch breaks at work, or, if they could not be active outdoors, they would find indoor spaces where they could walk. Parents reported that they started to walk more in general, engaged in activities such as skipping or skating, or conducted their own exercises in their homes. As a result of engaging in higher PA levels, parents noticed that they also experienced more positive mental health than before their involvement in the program. Parents explained that the changes that they implemented became established habits for them, and that these changes translated to them feeling more positively about other aspects of their lives.

Impact on family nutrition: It was reported that intervention group families’ nutrition behaviours improved through parents’ involvement in this program. Parents shared that their family planned for and made meals together thereby motivating their family not only to eat healthier, but also understand the importance of doing so. Parents explained that, because their family changed their diets together, they were more likely to engage in healthy eating due to the support they felt from each other. They noted that their children’s preferences for unhealthy snacks began to change, and they started to request fruits and vegetables. In addition, parents shared that their children who were not participating in the study also changed their diets and began to seek out healthy food options. Parents described that they continued to change their home food environments by ensuring their homes no longer had convenience or junk foods. They no longer bought fast food or snacks that were high in sugars or fats (e.g., granola bars); instead, they made fruits and vegetables more readily available in their homes.

Increase in family PA: Intervention group parents shared that because they began to increase their own PA levels, their family did the same. They explained that their involvement in the program helped them make PA a priority for them and their families. They started to engage in PA together, which soon became part of their routine. Some families went for walks together, whereas others set designated times to be physically active together. Parents noted that this increase in family PA was a drastic change from their previous habits in that they and their families would have been engaging in more sedentary behaviours prior to participating in the program.

Modeling behaviours: Participating parents in the intervention group shared that the program helped them realize the extent to which their behaviours impact their whole family’s behaviours. Parents explained that when they changed their behaviours, their children noticed, and did the same. Parents also felt it was important to establish healthy behaviours to ensure that their children developed healthy habits which would be more likely to continue over their lifetime. Parents reflected that they did not realize the extent to which their children observed their parents’ behaviours, and through the webinars and coaching program, parents were able to make changes that benefitted themselves and their families.

Overall program experiences: Parents described that through the intervention program they increased their motivation to engage in healthy behaviours, felt the program was positive overall, explained the impact of the webinars, and also provided their feedback regarding future directions and improvements. Children and families began to increase PA levels and improve dietary intake upon observing these behaviours being implemented by participating parents, thereby further motivating parents to continue improving their habits. In general, parents felt the program was a positive experience and provided the momentum they needed to institute and maintain healthy behaviours. Most liked the format of the program (i.e., telephone sessions and at-home follow-ups), and others were pleased to observe positive impacts on their children’s health behaviours. Some parents felt the education sessions provided them with some new information, while others felt they served as reminders of health information they already knew. Participants suggested providing printed copies of the webinars, using a different site to host the webinars, and more structured deadlines for webinar completion dates. In addition, it was suggested that the program be offered in schools, and because of their positive experiences with coaching, parents recommended that all participants in the program should have the opportunity to work with a coach.

Control group themes: Twelve themes, and 21 sub-themes, emerged from post-intervention interviews with parents in the control group (*n* = 13) regarding the impact of the program on themselves and their families. Nine themes and 16 sub-themes were unique to this follow-up time, and therefore are discussed in detail. Corroborative quotations for each new theme/sub-theme can be found in Table 10.

Increased awareness: Unique to this follow-up time, parents noted that their children who participated in this program increased their own awareness regarding healthy habits. Children, it was reported, noticed changes in their parents’ behaviours, and also started to make more mindful, healthy food choices as a result of seeing their parents do the same. Parents explained that they were not aware of the extent to which children noticed and engaged in the same behaviours as their parent.

Impact on child’s nutrition: Control group parents described that the impact of the program on their child’s nutrition behaviours varied. Some noted that their child’s nutrition improved in that fast foods and treats were limited, more fruits, vegetables and salads were introduced into their child’s diet, and food substitutions were made. For instance, one parent described substituting flour with oatmeal and bananas in pancakes. Many parents described that these changes were made without their child noticing. Parents also began to explain healthy eating to their child, and the importance of making healthy choices. Other parents described that the program had no impact on their child’s nutrition habits, because they felt their child already had a healthy diet.

Impact on family’s nutrition: The participating parent’s involvement in this study was perceived to impact family dietary intake behaviours. They described that family meals now included healthier options (such as salads), and that unhealthy snacks were being switched out for foods with low sugar and carbohydrate content. In addition, parents taught family members about portion sizes, and children’s lunches were prepared at the beginning of the week, and thus were better balanced than before the program. It was also expressed that because families had many, varying food preferences, change was hard to implement. Parents described that their partners were content with current food practices, or that their children had specific food preferences. In those cases, instead of preparing a separate meal for themselves, parents tried to include healthier alternatives to their families’ meals.

Impact on child’s PA: Parents described that their own involvement in the education program had an impact on their children’s PA levels because parents encouraged their children to become more active, which in turn caused their sedentary and screen time to decrease. It was explained that children no longer came home from school and sat on the couch, but instead went outside to play. Some parents enrolled their children in more activities or participated in PA with their child. Children enjoyed using the pedometers for this study, as they liked observing increases in their step counts, and also engaged in competitions with their parent who participated in the study. That said, some parents felt their children already engaged in high levels of PA, and as such there were no impacts on their children’s PA.

Impact on family’s PA: Parents perceived that their involvement in this program resulted in their family also experiencing health behaviour improvements. As noted at mid-intervention, families increased their PA levels and PA became more frequently done together compared to before the program. Parents explained that they engaged in more activities together such as walks, swimming, snowshoeing, and going to parks. In general, the family became more active as the parent increased their own PA and encouraged their family’s participation. However, some families did not experience any impact on their PA levels from the program. Parents felt that they and their family were already active, and thus nothing changed, or that they and their family discussed and reviewed information from the education sessions, but no changes had been implemented before the end of the current study.

Impact on own nutrition: Parents spoke about changes they made in their own diets, and how they made substitutions for different foods, or cut other foods out completely. They described that they and their families no longer consumed convenience foods, they packed healthy lunches, and they substituted healthier alternatives (e.g., apple with cinnamon) to snack foods (e.g., sweets or chips). Some parents explained that they used leftovers for other meals (e.g., lunches) more frequently, and others implemented stricter portion sizes for themselves. Conversely, other parents did not experience any program impacts on their diets, though some believed they became more aware of the food choices they made.

Impact on own PA: Many parents described that they felt their PA increased throughout the duration of this study. Some, in particular, made more conscious efforts to incorporate PA into their days. They described parking further away from their destinations, taking walking breaks at work, and using the stairs instead of elevators. Some parents explained that in trying to increase their child’s PA, their own PA increased as well. A few parents felt that their PA remained consistent over the course of the program and though they had intentions of increasing their activity levels, no changes had been made yet.

Modeling behaviours: Parents in the control group indicated that throughout the program, as they changed their behaviours and their home environment, their families’ behaviours changed as well. The parents who participated in the study noted that changes occurred in their partners and in their children because they themselves were making and modeling changes as a result of their involvement in this study. Some partners made changes in support of the parent who participated while others seemed to be motivated by the changes their partner was making. It was described that because the parent who participated chose to make changes in terms of food purchases, snacks, family meals, and PA, the rest of the family followed. Children, it was reported, noticed changes in their parents’ behaviours, and also started to make more mindful, healthy food choices as a result of seeing their parents do the same. Parents explained that they were not aware of the extent to which children noticed and engaged in the same behaviours as their parent.

Accountability: Parents in the control group described that participating in this program increased their accountability for their own and their families’ habits. They explained that by logging their dietary intake, they were able to review the foods they ate and felt an increase in accountability to make better choices. In addition, the in-person follow-ups were a source of accountability in that parents felt they should make healthy choices because they perceived the research team would be checking in on them at various time points.

Motivation: Participants described varying experiences regarding motivation. Some parents noted that their participation in the program motivated them to make changes in their health behaviours. They explained that the information from the program was the catalyst they needed to change their habits. Healthy behaviours became a priority for some in that they were more motivated to increase their PA or make better health decisions overall, compared to before the program. Conversely, other parents reported ambivalence toward behaviour change. They were aware of changes they felt they needed to make, and had ideas of how to do so; however, they did not yet feel motivated to implement them. Others explained that though they started to make changes, when they faced set-backs they were unmotivated to start over or continue.

Impact of program: Unique to this follow-up time, parents shared that they had a positive experience overall, in that they liked that the program incorporated their children’s participation, appreciated that follow-ups were conducted in their homes, and felt it provided them with the push or reminder they needed to make changes in their lives.

#### 3.2.3. Six-Month Follow-Up Themes

Intervention group themes: Seven themes and 11 sub-themes were identified from six-month post-intervention interviews with intervention group parents (*n* = 9). Two themes and eight sub-themes were unique to this follow-up time, and therefore are discussed in detail. Corroborative quotations for each new theme/sub-theme can be found in Table 11.

Overall impact of coaching: During their six-month follow-up interviews, parents in the intervention group again stated that coaching was a positive experience for them. They explained that through coaching, they realized the interconnected nature of physical and mental health allowed them to better understand the process of their own behaviour change. In addition, parents also recounted that coaching made them feel accountable and motivated to change their behaviours, and even after coaching concluded, they continued making changes. They explained that this differed from their previous behaviour change attempts in that, with added stress, they normally would have let their own health falter. Parents restated that coaching allowed them to work through root causes of their behaviours, which in turn improved their mindset, and helped them alter the way they viewed behaviour change. Parents shared that coaching had lasting impacts in that they continued to use strategies provided by their coaches such as journaling, identifying roadblocks, and prioritizing themselves.

Impact on own and family’s nutrition: Unique to this follow-up time, parents explained that implementing meal preparation into their routines facilitated healthy eating in their homes and made the healthy choice the easier one. When parents and their families were aware of what was available in their homes, and what they were preparing for each meal, they were less likely to seek out convenience foods.

Barriers to behaviour change: Parents in the intervention group shared that they faced barriers to behaviour change during the six months following program completion, these included: (a) stress; (b) motivation; and (c) time. Without the support of their coaches, some parents reported the changes they implemented had reverted back to their old habits. Some noticed that with increased stressors, they returned to using unhealthy or convenience foods for comfort. Others explained that their ambivalence toward behaviour change had reoccurred; however, they were more aware of their excuses that hindered them from making changes. Due to their children’s busy schedules, parents felt they lacked time to engage in their own PA. In addition, parents faced other barriers to improving their and their children’s PA and nutrition habits, such as poor weather and increasing prices for healthy foods. With their children transitioning to full school days, some parents reported that their children were more sedentary at school than they were at home. Parents also noticed that their friends and other family members (e.g., their own parents) made committing to behaviour changes difficult because the need to change diets or cut out certain foods was not understood by others.

Control group themes: Eight themes and 18 sub-themes were identified from six-month follow-up interviews with control group participants (*n* = 10). Two themes and six sub-themes were unique to this follow-up time and are therefore discussed in detail. Corroborative quotations for each new theme/sub-theme can be found in Table 12.

Prevention: A prominent theme at six-month follow-up was that parents in the control group further realized steps they needed to take to prevent their children from inheriting less healthy habits and prevent the development of adverse health effects associated with overweight/obesity in themselves; Unlike other follow-up times, at six-month follow-up, many parents in the control group explained that they realized the long-term impact overweight/obesity could have on their lives (i.e., diabetes, physical limitations, joint pain), which motivated them to make changes to their PA levels and dietary intakes. They stated that they did not want physical limitations to prevent them from engaging in activities with their families, and that it would be vital to implement changes at this point in their lives (i.e., before the onset of ailments). Lastly, participation in this program encouraged parents to explain the importance of healthy choices to their children to ensure that healthy food habits would persist into their later life stages.

Change in perspective: In making improvements to their PA and dietary intake, parents noticed changes in their perspectives. They explained that they put less emphasis on weight loss, and more on developing lasting lifestyle changes toward healthy behaviours overall. This investment in their own health behaviours resulted in some parents prioritizing themselves by ensuring that they set time aside for their own PA and self-care activities. Changes in mindset facilitated behaviour changes in parents because when they felt better about themselves, they were more likely to engage in PA or eat well, further motivating them to continue improving their health behaviours overall.

Barriers to behaviour change: At six-month follow-up, parents in the control group reported barriers that prevented them from continuing behaviour change after their post-intervention follow-ups. Unique to this follow-up time, and similar to the intervention group, some parents in the control group also experienced the return of their ambivalence. They described that they knew they should be engaging in PA and consuming healthy foods, but their motivation to do so had tapered. Given that seasons had changed over the duration of program follow-ups, many participants described that they felt less inclined to engage in activities when it was raining or snowing. Lastly, some parents spoke about other barriers including social situations (i.e., frequent socializing was associated with eating at restaurants), and the accessibility of junk foods when grocery shopping.

## 4. Discussion

Quantitative data varied at each time point (i.e., baseline, mid-, post-, and six months post-intervention) and between groups. Decrease in sodium intake from baseline to six-month follow-up showed a trend toward significance (*p* = 0.04) in parents in the intervention group. While the data were not statistically significant, trends in data are important to note for future research and are addressed below.

Similar qualitative themes were identified between intervention and control groups at all follow-up time points (i.e., mid-, post-, and six months post-intervention), with the intervention group reporting more salient experiences with: goal setting; accountability; addressing root causes of behaviours; changing perspectives; and improving mindset. Coaching provided the intervention group with new, lifestyle skills and perspectives not reported by the control group, though it seemed both groups experienced similar barriers at the six-month follow-up. Also of interest is that the control group perceived no impact of the program on their, their child’s, and their family’s behaviours more frequently than the intervention group. However, at six-month follow-up, the control group expressed wanting to change their behaviours in order to prevent the same behaviours in their children and the consequent development of adverse health conditions, while the intervention group did not.

Compared to baseline, children in the intervention group increased their step counts at all time points. Interestingly, children in the control group recorded high step count at baseline, and their step count remained around this level for the duration of the program. Given that most parents in both groups described their children as highly active before the program began, major increases in their children’s step counts may not have occurred as a result of the program. In their interviews, parents noted that they began to take more notice of their children’s PA behaviours, thereby prompting them to encourage increased PA in their children. In addition, many parents in both groups described that their children thoroughly enjoyed using the pedometers to track their step counts, which in turn motivated them to accumulate more steps. This may have resulted in step counts that were higher than normal for some children in this study. Parents explained that their families started to engage in more PA together, and some noted that their children developed a habit of being physically active after school as opposed to their pre-program sedentary behaviour.

According to CSEP (Canadian Society for Exercise Physiology) [7], adults who achieve greater than or equal to 7500 steps per day are classified as having a ‘physically active lifestyle,’ and those who attain 5000 to 7499 steps per day fall in the ‘low active lifestyle’ category. Currently in Canada, 52% of adults are considered to have a ‘physically active lifestyle,’ while 29% engage in a ‘low active lifestyle.’ Based on these guidelines, adults in both intervention and control groups were classified in the ‘low active lifestyle’ from their baseline step count. Following the program both groups improved to the ‘physically active lifestyle’ category at post-intervention. At six-month follow-up, the control group continued to increase step counts. Parents in the intervention group recorded fewer steps at six-month follow-up than their respective baseline mean. Interestingly, both groups had increased their active MET minutes at mid-intervention, when compared to baseline. It could be deducted, when comparing quantitative and qualitative findings, that immediately after joining the program parents felt motivated to increased their PA. Parents in the control group decreased their sitting time per day at all time points. At six-month follow-up, parents in the intervention group had higher sitting time than they did at baseline. Many parents described their jobs as involving high amounts of sedentary time, in that they worked at desks and found it difficult to take breaks during the day. However, parents also reported that their involvement in the current study encouraged them to take more deliberate stretching and walking breaks during their days.

Children’s dietary intake did not change significantly during or following the program; small fluctuations in intake of proteins, fiber, saturated fat, and sodium were reported. It is of interest to note that changes in children’s diet did not reflect those of their parents’. Hammersley, Okely, Batterham, and Jones [62] conducted a parent-focused, internet-based healthy lifestyle program for preschool-aged children who have overweight/obesity or were at risk for developing the condition. Using a randomized controlled trial (RCT) design parent-child dyads were randomized to receive either an 11-week healthy lifestyle program and sessions with a dietitian (intervention group), or email communication only (comparison group) [62]. The researchers measured child BMI, physical activity, parent-reported dietary intake, screen time, child feeding, parent modeling, and parent self-efficacy at baseline, three months, and six months [62]. The researchers found no significant differences for change in BMI between groups, however dyads in the intervention group showed a reduced frequency of discretionary food (e.g., takeout, fast food, sugary foods, salty snacks etc.) intake, and parents showed improvement in child feeding pressure to eat practices, and nutrition self-efficacy [62]. The findings fromref. [62] and the current study suggest that having support when implementing behaviour change may result in greater uptake of these changes.

While there were no major changes in children’s dietary intake in the current study, parents reported that they perceived their children’s awareness of healthy foods increased through their involvement in this study. Parents reported that children became more cognizant of their dietary intake choices and what foods were considered ‘healthy’ versus ‘unhealthy.’ Through their parents’ involvement in this study, children learned how to interpret nutrition tables, and made more informed nutrition choices. In a comparable study, following a highly participatory, community-based PA and nutrition intervention for children (ages 6–10) and their families, children had decreased their energy, fat, saturated fat, carbohydrate, and sodium intakes [63]. This would suggest that including more interactive child-based components in the current study may have resulted in more significant changes in children’s nutrition. At baseline, parents in both groups consumed well over the recommended amount of sodium (2300 mg per day [64]) for Canadians. In order to avoid health risks (e.g., hypertension), it is recommended that individuals should not exceed consuming the recommended amount of sodium per day, yet approximately 80% of Canadians intake 3400 mg of sodium per day [65]. Parents in the intervention group substantially decreased their sodium consumption to below 2300 mg, as is evident in the interaction plot. Although this decrease was not statistically significant (*p* = 0.04), it is important to note that action toward decreasing unhealthy behaviours was being implemented. This decrease in sodium consumption could be attributed to parents having a better understanding about the information on nutrition labels, and/or, limiting processed or convenience foods in their homes. Some parents reported that by reducing their snack or convenience food intake, their cravings for such foods also dissipated. Limiting these foods might have subsequently decreased their sodium consumption. Sodium intake of children in the intervention group did not reflect the same pattern as their parents’ intake. At baseline, children in the intervention group were consuming slightly less than the recommended amount of sodium, whereas at six-month follow-up they were consuming more than the recommended amount.

At six-month follow-up, the intervention group had a slightly lower BMI and waist circumference compared to baseline. Parents in the control group did not experience many changes in BMI. In a 12-week pre-post, study, Goddard and Morrow [66] aimed to determine the impact of telephone-based motivational interviewing via CALC (MI-via-CALC) on PA engagement, BMI, and hip and waist circumferences in Canadian women ages 30–55. While the researchers reported no statistically significant difference in PA engagement, they noted that 16 of their 19 participants achieved weight loss, resulting in significant BMI reductions over the course of the intervention [66]. These downward trends have been noted in previous studies in which the researchers found that individualized CALC interventions can produce significant BMI reductions [41,66,67]. For instance, Pearson and colleagues [38] assessed the effectiveness of two self-management approaches on obesity via a 12-week telephone-based intervention in which one group received MI-via-CALC while the other received a structured lifestyle program. Participants receiving MI-via-CALC decreased their BMI and caloric intake—with the majority of reductions in fat versus carbohydrate or protein-based calories—between baseline and week 12 [37]. Further, Newnham-Kanas, Irwin, Morrow, and Battram found that their participants, adults with obesity (*n* = 8; ages 35–55), also experienced weight loss and reduced waist circumference after receiving MI-via-CALC for 18 weeks [68]. Though the current study did not show similar significant changes in BMI, the aforementioned studies demonstrate that CALC can impact body composition. In addition to a small sample size, it is possible that the six-month follow-up period in the current study was not a long enough duration to observe statistically significant changes in body composition. A longitudinal study would allow for assessment of long-term changes in participants. In addition, a longer follow-up period (>6 months) might allow for more significant changes in PA and nutrition behaviours, which, in turn, might result in decreased BMI. It has been noted that in the absence of a weight maintenance program, weight loss tends to reverse with 50% of participants returning to their original weight after five years [69]. Researchers have noted that employing extended care models in obesity treatment studies (e.g., text message reminders, in-person group sessions) have resulted in increased weight loss maintenance, and long-term PA and dietary behaviour change [69]. This is because extended care models provide patients with the support and motivation needed to continue implementing behaviour changes [69]. Many participants in the current study expressed feelings of ambivalence at six-month follow-up, suggesting that a form of extended care might have assisted in continuing behaviour changes they implemented during the program [69].

Parents in the intervention group explained that changing their health behaviours (e.g., increased PA, and healthy food choices) resulted in them developing positive mental health, thereby motivating them to maintain those changes. Those in the intervention group expressed the desire for a program that would have allowed them to work through their psychological challenges to change (e.g., sources of ambivalence toward behaviour change). Program benefits (e.g., accountability, goal setting skills, identifying root causes of behaviours, change in perspectives) were more salient in responses from parents in the intervention group than those in the control group. In a 12-week obesity treatment study for university students with obesity, it was noted that while both groups benefited from their involvement in the study, the control group (who received education) gained insights into practical aspects of behaviour change, whereas the intervention group (who received CALC) were able to focus on underlying causes of their behaviours [70]. These findings are consistent with previous research which states that, while education is effective in changing knowledge toward health behaviours, education alone is insufficient in changing behaviour [43,44].

Researchers have noted that emphasizing weight-related measures in obesity studies may unintentionally draw parents’, and consequently children’s, attention to weight; thus, foci should be shifted towards the more positive notion of healthy lifestyles [69]. Parents in both groups explained that through the use of pedometers and food tracking, they increased their awareness of their habits, and the webinars provided them with information to help modify their behaviours. They expressed that learning about various aspects of nutrition (e.g., reading nutrition labels) and PA (e.g., recommended daily levels for each age group) motivated them to make changes not only in their own lives, but also in their families’. Ref. [71] reported that mothers of preschoolers (2–5 years old) who received health information via mail and 20–30 min telephone coaching sessions incorporating motivational interviewing, over the course of eight months, reduced sugar-sweetened beverages, increased fruit and vegetable consumption, and changed their home environment (e.g., fewer meals eaten in front of television) for them and their children. These findings are consistent with findings from both control and intervention groups in the current study. It has also been found that attempts to improve healthy lifestyles in the home can be more effective if parents are able to adopt and model these behaviours themselves [71]. Again, parents in both groups of the current study reported that modeling healthy behaviours resulted in their children wanting to do the same.

It could be speculated that because parents in the intervention group were no longer working with their coaches, they experienced a slight decline in behaviour changes they had implemented during the program. In their interviews, parents in the intervention group reported that coaching allowed them to work through root causes of their behaviours and frequent roadblocks they faced when attempting to implement changes. In addition, parents also reported wanting more coaching sessions to work through more of their behaviours. Given that Co-Active coaching centers on encouraging the client to set the agenda for sessions, parents in the intervention group spent some of their sessions working through these issues. Thus, it is possible that with more coaching sessions they may have made more significant, targeted behaviour changes.

It has been reported that increases in autonomous motivation (AM), the most self-determined form of motivation, predicts improvements in food choices and long-term adherence to PA in both men and women [72]. Conversely, controlled motivation measures the extent to which a person feels external pressures to change, and is associated with low adherence to behaviour change [52]. In a study of a national sample of parent-adolescent dyads (of any weight), it was found that increased AM was positively correlated with fruit and vegetable intake in that high levels of AM were associated with greater fruit and vegetable intake [73]. In the current study, AM did not vary greatly within or between groups with regard to diet or exercise; both groups reported high AM throughout the program. It could be argued that, given that motivation was high at all time points, this group of parents were already highly motivated to make changes in their behaviours (which may have been exemplified by their decision to join the study); therefore, any changes in motivation would have been minimal [73]. Similarly, in the current study, parents reported feeling more motivated to make behaviour changes when they perceived feeling better about themselves. Fisher and colleagues [74] explored how proficient parents were at setting health behaviour goals for their children, and examined collaboratively set goals (i.e., in a group setting) compared to self-determined goals. They found that parents who set self-determined goals had significantly higher goal setting scores than those who created collaborative goals [74]. The researchers explained that intrinsic value of a goal is a significant predictor of goal setting and subsequent behaviour change in adults [74]. In their interviews, parents from both groups in the current study described developing increases in motivation to engage in PA and consume healthier foods; however, parents in the control group reported higher levels of AM at six-month follow-up when compared to baseline. This finding is surprising in that at six-month follow-up parents from both groups explained that they began to experience more ambivalence toward behaviour change in the face of barriers upon program completion. Motivation has been reported as being high in the initial stages of a lifestyle intervention, whereas maintaining motivation is challenging [74]. Burgess et al. [75] explored barriers to behaviour change for adults with overweight/obesity, which included: lack of time; environmental, societal, and social pressures; health and physical limitations; and difficulty managing negative thoughts or moods [75]. Parents in the current study explained a few similar barriers (e.g., lack of time) that prevented or discouraged them from maintaining their behaviour changes. It can be inferred from these findings that some parents require assistance with maintaining their motivation to make healthy choices upon completion of behaviour change programs.

### 4.1. Strengths

Various techniques were employed to ensure study quality and credibility of data. First, inclusion of a qualitative component, through a mixed-methods study design, enhanced external validity of the RCT [40]. Parent interviews allowed researchers to understand and contextualize changes in the outcomes that were not evident in quantitative findings. From the qualitative component, the researchers gained insights into the impact of the program on the participants that were not reflected in quantitative results. In addition, including negative cases (i.e., feedback that is not consistent with positive findings; [76]) provides researchers with critical information regarding aspects of a program that may not be best suited for a particular group, or illuminates areas for program improvement (e.g., having webinars available in audio format. Conducting interviews at multiple time points also allowed researchers to assess any changes in experiences over the course of, and following, the program. This study may have been the first to incorporate a process-oriented approach [77] to explore experiential changes over time. Highlighting themes that were the same or different at the various time points reveals process-oriented changes, particularly when quantitative data did not provide robust results. Employing a rigorous qualitative component over time can be valuable in understanding how experiences changed over the duration of the program and follow-up periods. The utilization of a standardized coaching method allowed for participants to receive similar models and techniques of coaching, reducing variability of experiences. The webinars greatly influenced the control group, more so than anticipated, resulting in both groups qualitatively reporting substantial changes in their environments and behaviours. Some parents in the control reported benefitting greatly from the webinars alone, and made important changes in their lives due to their program experiences (e.g., meal preparation; prioritizing themselves).

### 4.2. Limitations and Future Directions

Despite the research team’s efforts to recruit both fathers and mothers, the majority (*n* = 47) of participants were mothers. In a systematic review conducted to measure participation rates of fathers in obesity treatment and prevention programs targeting children and adolescents ([78]; 0–18 years); the researchers reported that of the RCTs that limited participation to one parent only (*n* = 80), fathers only represented 6% of parent participants [78]. Davison and colleagues [79] examined the representation of fathers in family interventions to prevent childhood obesity, and also found that only 6% of parents who participated in the eligible studies (*n* = 30) were fathers. Interestingly, in the current study only three of 50 parent participants were fathers, a fact consistent with the aforementioned 6% participation rate. Fathers impact children’s weight-related behaviours during early childhood, such as diet, PA, and media use [79]. Thus, it seems imperative to recruit actively and perhaps even target fathers in obesity treatment and prevention interventions [80,81].

Recruitment and retention of participants also served as a limitation in this study. Although approximately 300 different organizations were contacted to assist with recruitment efforts, the target sample size was not obtained until 15 months after recruitment began. In order to ensure the program was convenient for participants, the intervention was offered over the phone and online, and the research team conducted follow-up assessments at participants’ homes. Despite this, retention of study participants also posed difficulty, in that participant dropout occurred at each follow-up point. Researchers have reported that participant recruitment and retention in childhood obesity interventions, particularly in community settings, pose challenges due to the time required for baseline measurements, intervention delivery, post-intervention follow-ups, and sustainability measures [82]. It is possible that deterrents for participation in the current study included the intensive nature of the program (e.g., questionnaires, in-person assessments, several follow-up times, food and step tracking). A few parents in the intervention group did not complete their coaching sessions; as coaching is a highly personal experience, and immediately targets sensitive topics, it is possible that these participants did not enjoy the nature of the intervention. This can be inferred from parents who did complete coaching, who explained that coaching felt awkward at the outset but improved over the duration of their sessions. Given that participants were randomized to treatment, it is possible that the fit with their coach was not natural for them. In the future, offering a self-selection option may be valuable in order to determine whether this impacts adherence to treatment.

The lack of statistically significant quantitative results may be due to attrition and heterogeneous group difference shifts, meaning that some individuals may have experienced stronger impacts from the program than others but the group itself did not. Given that coaching is an individual experience, it is not surprising that the intervention effected individuals differently. Attrition was high at the end of the follow-up times, thus preventing any statistically significant results due to lack of power. In addition, while the researchers hoped to explore the impact of the program on dietary intake by analyzing nutrients, changes were very subtle and varied between individuals and groups. In order to observe changes at the nutrient level, a larger sample size is needed. In general, the small sample size did not allow for an in-depth assessment of some outcomes. In addition, self-report bias and recall error may have resulted in less accurate data compared to direct measures [83]. Social desirability bias (i.e., answering questions in a manner that will be viewed positively by the researchers; [84]) may also explain differences in qualitative and quantitative data, in that participants may have been overstating changes in their behaviours.

## 5. Conclusions

Although, from interview findings, it seemed that both control and intervention groups experienced similar benefits from program involvement, the marked difference, based on qualitative results, was goal setting, accountability skills, and improved mental health in the intervention group. The intervention group was able to work on identifying and addressing root causes of their behaviours. While the program enabled both groups to heighten their awareness and education of health behaviours, coaching allowed parents to select issues in their lives they wanted to work on, which, in turn, helped them improve their PA and dietary intake behaviours. Based on feedback from parents, coaching appears to be an effective method to initiate behaviour change; however, it seems participants require a form of extended care to assist them with maintaining positive changes. The control group benefited from the webinars but wanted more external support for behaviour change. Future research should explore the impact of more frequent and/or longer coaching sessions, as well as integrate more participatory methods for child-participants (e.g., inclusion in coaching sessions), and measures (e.g., interviews or surveys) to gain their perspectives on changes over time.

## Figures and Tables

**Table 1:** Interview Questions.

- Please give us feedback on this program, positive or negative, about your experiences so far
○ Please elaborate on what has assisted with behaviour change and what has not
- What did you like best about the program?
○ What parts of the program did you find most helpful, and why?
- What did you not like about the program?
- Thinking back to before you started the program compared to now:
○ What impact do you think the program has had on the physical activity behaviours of your child (the one who is registered in the study)?
○ What impact do you think the program has had on your physical activity behaviours?
○ What impact do you think the program has had on the physical activity behaviours of your family, as a whole?
○ What impact do you think the program has had on the dietary intake/nutrition of your child (the one who is registered in the study)?
○ What impact do you think the program has had on your dietary intake/nutrition behaviours?
○ What impact do you think the program has had on the dietary intake/nutrition behaviours of your family, as a whole?
○ What would you say is the most important thing you learned from being in the program?
○ If we were to provide this program again, what recommendations would you have for any changes we should make?
○ What else do you want us to know about your experience with the program and how it has influenced you, your child, and your family?

**Table 2:** Demographic Characteristics of Parent-Child Participants.

Participant Characteristic (Baseline)	N	%	Mean	SD
Parent Sex				
Male	3	6		
Female	47	94		
Parent Ethnicity				
Caucasian	43	86		
African Canadian	2	4		
Latin-American	2	4		
Asian	1	2		
Other	2	4		
Parent BMI (kg/m^2^)			36.1	7.3
Parent Waist Circumference (inches)			44.0	5.6
Parent Education (Highest level completed)				
Secondary/High School	6	12		
College	20	40		
University	17	34		
Graduate School	7	14		
Family Situation				
Single-parent	8	16		
Double-parent	42	84		
Number of people in household				
2	0	0		
3	12	24		
4	26	52		
5	6	12		
6	5	10		
7 or more	1	2		
Annual Household Income				
Less than $20,000	1	2		
$20,000–$39,999	7	14		
$40,000–$59,999	5	10		
$60,000–$79,999	10	20		
$80,000–$99,999	4	8		
$100,000–$119,999	3	6		
$120,000–$149,999	10	20		
>$150,000	5	10		
Prefer not to answer	5	10		
Child Sex				
Male	18	36		
Female	32	64		
Child Age			6.8	2.8
Child Ethnicity				
Caucasian	39	78		
African Canadian	4	8		
Native/Aboriginal	1	2		
Latin-American	2	4		
Asian	1	2		
Other	2	4		

**Table 3:** Child Nutritional Variables and Step Count for Intervention and Control Groups, at Baseline, Mid-Intervention, Post-Intervention, and 6-Month Follow-Up.

	Intervention Group Baseline	Intervention Group 6-Week	Intervention Group Post	Intervention Group 6-Month	Control Group Baseline	Control Group 6-Week	Control Group Post	Control Group 6-Month
Mean steps (SD)	6830 (1782.55)	10,371 (6884)	11,179 (4323)	9259 (2072)	11,671 (4264)	10,741 (3520)	11,016 (4700)	10,537 (4981)
Protein, g (SD)	59.5 (17.3)	64.1 (25.1)	75.3 (24.2)	50.2 (10.8)	75.9 (23.5)	67.8 (23.4)	69.7 (20.9)	71.8 (14.8)
Fibre, g (SD)	17.7 (13.1)	15.9 (6.5)	15.6 (3.8)	15.2 (8.1)	17.9 (9.2)	15.9 (7.8)	17.7 (7.8)	18.9 (6.1)
Saturated Fat, g (SD)	17.9 (5.5)	25.7 (14.5)	17.2 (8.0)	23.9 (26.0)	21.4 (8.8)	16.7 (10.9)	20.7 (16.6)	26.5 (16.1)
Sodium, mg (SD)	2204.1 (806.3)	2467.0 (1498.3)	2119.9 (608.9)	2848.1 (1874.7)	2551.9 (1016.26)	2306.7 (2113.08)	2904.1 (2275.6)	2698.2 (1343.7)

**Table 4:** Parental PA & Anthropometric Variables for Intervention and Control Groups, at Baseline, Mid-Intervention, Post-Intervention, and 6-Month Follow-Up.

	Intervention Group Baseline	Intervention Group 6-Week	Intervention Group Post	Intervention Group 6-Month	Control Group Baseline	Control Group 6-Week	Control Group Post	Control Group 6-Month
Mean steps (SD)	6381 (1731.8)	7396 (2531)	8700 (6053)	5871(1341)	6550 (2726)	6805 (2270)	7677 (1896)	10,331 (5021)
Mean BMI (SD)	36.7 (7.6)	37.3 (8.4)	36.7 (8.4)	34.8 (7.8)	35.8 (7.3)	36.5 (8.5)	35.9 (9.6)	36.8 (8.9)
Mean Waist Circumference, inches (SD)	44.1 (6.2)	43.5 (6.4)	43.8 (7.2)	42.6 (6.9)	43.7 (5.6)	43.5 (6.2)	42.9 (7.1)	43.9 (6.6)
IPAQ MET Mins Per Week (SD)	1113.6 (1009.6)	1894.8 (1600.0)	1603.8 (773.3)	1436.4 (347.1)	1948.4 (1451.1)	2920.2 (2084.5)	2044.5 (1095.2)	2394.0 (1497.1)
IPAQ Sitting Mins Per Day (SD)	261.8 (181.3)	580.0 (1340.8)	240.0 (144.7)	285.0 (79.9)	354.6 (247.8)	328.9 (210.8)	326.4 (108.8)	222.0 (176.8)

**Table 5:** Parent Nutritional Variables for Intervention and Control Groups, at Baseline, Mid Intervention, Post Intervention, and 6-Month Follow-Up.

	Intervention Group Baseline	Intervention Group 6-Week	Intervention Group Post	Intervention Group 6-Month	Control Group Baseline	Control Group 6-Week	Control Group Post	Control Group 6-Month
Calories (SD), kcal	2026.5 (753.3)	1833.9 (1741.7)	2012.8 (622.3)	1810.5 (730.3)	2256.8 (557.9)	1884.3 (561.2)	1741.7 (438.4)	1660.2 (381.4)
Protein (SD), grams	82.1 (34.8)	86.6 (34.8)	107.1 (37.9)	70.7 (31.9)	98.1 (37.3)	94.7 (31.3)	88.4 (22.9)	98.9 (33.9)
Fibre (SD), grams	24.1 (18.2)	24.7 (17.5)	20.8 (5.7)	19.5 (7.8)	22.6 (10.4)	23.6 (11.1)	18.6 (9.7)	17.1 (7.5)
Saturated Fat (SD), grams	24.8 (12.4)	27.6 (23.2)	27.4 (14.9)	30.7 (25.7)	34.0 (17.1)	21.9 (12.1)	33.4 (26.7)	28.5 (17.7)
Sodium (SD), milligrams	3380.8 (1790.3)	2670.5 (2000.8)	2617.9 (1332.0)	1942.9 (1450.6)	3659.4 (1713.3)	2450.2 (1657.7)	2766.4 (1571.7)	2468.9 (1737.0)

**Table 6:** TSRQ Variables for Intervention and Control Groups, at Baseline, Mid Intervention, Post-Intervention, and 6-Month Follow-Up.

	Intervention Group Baseline	Intervention Group 6-Week	Intervention Group Post	Intervention Group 6-Month	Control Group Baseline	Control Group 6-Week	Control Group Post	Control Group 6-Month
TSRQ Diet, Autonomous Motivation (SD)	5.9 (1.0)	5.9 (1.1)	5.6 (1.2)	5.6 (1.2)	6.0 (0.8)	5.9 (0.8)	5.7 (0.9)	6.2 (1.0)
TSRQ Diet, Controlled Motivation (SD)	3.8 (1.3)	3.9 (1.3)	4.0 (0.9)	3.5 (1.6)	3.7 (1.1)	4.6 (1.2)	3.8 (1.1)	3.6 (1.5)
TSRQ Diet, Amotivation (SD)	2.2 (1.0)	2.1 (1.2)	2.5 (1.0)	2.5 (1.4)	2.4 (1.1)	2.5 (1.4)	2.3 (1.2)	2.0 (0.9)
TSRQ Exercise, Autonomous Motivation (SD)	5.9 (1.3)	5.8 (1.5)	5.8 (0.9)	6.0 (1.0)	6.0 (1.0)	5.6 (1.0)	5.6 (1.1)	6.0 (1.3)
TSRQ Exercise, Controlled Motivation (SD)	3.5 (1.2)	3.8 (1.1)	3.9 (1.3)	3.6 (1.2)	3.6 (1.5)	4.2 (1.2)	3.8 (1.4)	3.6 (1.5)
TSRQ Exercise, Amotivation (SD)	2.1 (1.3)	2.2 (1.2)	2.6 (1.6)	2.9 (1.3)	2.1 (1.2)	2.6 (1.4)	2.4 (1.9)	1.7 (1.0)

**Table 7:** Corroborative Quotations for Mid-Intervention Themes and Sub-Themes (Intervention Group).

Theme: Increased Awareness
Quote: “…The coaching aspect has definitely [made] a big difference for me. Because I can read the module, and then for a day I really think about it, or the days where, you know, I have to check the steps, I’m much more conscious of it, of, ‘Okay, I’ve got to do this.’ I want [my children] to be active, you know, I want to be on it. Or when I had to write down my food, I thought about it a lot more.”
Theme: Modifying Own and Family Behaviours
Quote: “I think that my nutrition’s changing … in the options that I’m picking, ‘cause I have to eat certain amounts of carbs, but it’s trying to make those healthier options of what type of carbohydrates I’m eating. Like, having a piece of bread or vegetable or fruit instead. … Is a much better option than having a bowl of cheezies. … I have to have a snack every night I’ll have a peanut butter sandwich instead of a bowl of chips.”
Theme: Impact of Webinars
Quote: “The biggest [learning] … that was impactful for me was going into the grocery store and reading the ingredients a little bit more. … You see things like 20 percent or 5 percent, and it didn’t mean anything to me [before completing the webinars].”
“I really like some of the tips for handling the issues…with children and eating… The idea of not giving them food when they’re upset… was interesting to me and the way it was described [I] was like, ‘Yeah that makes sense.’ If you feed them when they’re upset then they learn that food just makes them feel good… I can see how that would lead to emotional eating.”
Theme: Barriers
Quote: “I think over the winter … some of [webinar] ideas will be good, because we’re kind of stuck inside now. … That’s where we kind of get … lazier. So I think just doing different activities inside and stuff will be really good.”
“[If] I went to the YMCA that’s closest to me now I’m gone for over an hour to go to a class that’s 30 min. … It’s just a lot to take out of your day when you have two young kids at home.”
“So, if you are buying a lot [of healthy food], that adds up. Same with every single fruit. … even the ones that are not organic [are] expensive. The meat went through the roof, certain spices [are] way … too expensive.”

**Table 8:** Corroborative Quotations for Mid-Intervention Themes and Sub-Themes (Control Group).

Increased Awareness & Motivation
● Pedometers
“This [pedometer] is addictive. … I’m surprised how many more steps [my daughter] does in a day. … She was at … 16,000 where I was still at [9000].”
“My daughter… is talking about [the program] a lot, and was very excited about the step counters, and was …Very excited to be like, ‘Hey, look at how many steps I’ve achieved.’”
● Eating Habits
“I have noticed … my daughter… is a lot more active than I am currently, but I have noticed…what we’re eating is starting to affect her. … my bad choices are now affecting the entire family, not just me … [A]ll of the [webinar] videos that were related to the child part of it … she watched with me. … She enjoyed that. But I think for me too, it’s realizing that the choices I’m making are affecting more than just me.”
“I have a job … driving around the city all day… [leading to] bad habits of going through drive-thrus… so I am now making more conscious decisions to stop at the grocery store, if I don’t have a lunch … I can just get a salad or something a little more healthier. … [The program has] made me more conscious that way.
● Sedentary Behaviour
“[My child] loves YouTube. … So, we’ve always limited …screen time. But it’s interesting, when [the webinar is] saying… one or two hours a day of screen time. You think about how that adds up so fast. … She’s eating her breakfast, she comes home, she watches a little bit. It can easily be an hour, two hours without thinking about it. … So that’s definitely something that… I pay attention to.”
Implementing Healthy Behaviours
“[My child] can be a little bit of a picky eater, so [prior to joining the program] we would end up just caving into him and giving him whatever he wanted. … So, we cut back on that.”
“My husband and I have … got rid of all treats in the house, so when it’s a snack … the only option available is fruit.”
“… [I am] parking further away and walking and just trying to get as many steps in as possible.”
Impact of Webinars
“The program itself has been good information. The videos are good …it wasn’t a lot of things that I didn’t already know. … But, it’s good to … have the reiteration of things that I should know, but don’t follow.”
“I read [daily value percentages] now every time I go to a grocery store, I always check the values, I check the fat, I check everything that is not healthy, I try to stay away from that. … [Before] I wouldn’t care about that much.”
Barriers
● Time
“[L]ife is busy, so… I kind of forgot about the [webinars] and so I have to remind myself to do that.”
● Family members
“I find it frustrating sometimes … because my husband doesn’t eat vegetables. … And so, I’ll try to prepare … carrots and make them a bit sweet, or try and … entice [my family] with it. … And my husband will be making a face ‘cause he doesn’t like it and I’m like, ‘Can we not? Just like, don’t influence [the children], right? Or don’t say you don’t like it, can you not just eat one carrot and smile?’”
● Stress
“I did start [eating healthy] for quite a while [during the program]. I was doing really well, and then some things changed, and there was a little bit too much stress…then I took a week off, and I realized how easy it is to fall back into bad habits.”
● Weather
“But when it is that hot [outside], most people are staying in and kind of hibernating, because it’s too hot to be out, and so we have been trying to get outside every single day, but even if it’s just the backyard, but again, walking from my bedroom to the back door isn’t really a lot of exercise…”
● Picky eaters
“I also feel like incorporating [healthy foods] in a way that [kids] don’t know isn’t really teaching them why it’s important [to eat healthy]. … And I also wish they would just give it a chance. Cause like, I make turnips, and I use a little bit of brown sugar, and I know that the oldest would like it, it’s just there’s no chance [my younger child will] even try it.”
Program Improvements
“I would forget about the online [webinars], just cause it’s online or whatever, maybe … a reminder… every week … Just kind of like touch base and remind you that the videos are on there.”
“… I think that extra accountability piece would have made a difference. Whereas, now this is the second time we’re meeting, it’s a couple months in, and… I know I’m accountable but not to the extent … if I was also getting that [coaching] phone call. I would have been, like, ‘Oh my gosh, they’re gonna know even more.’ … Not that I’m hiding anything, but… just having the extra little bit of accountability… would have been better in my situation.”

**Table 9:** Corroborative Quotations for Post-Intervention Themes and Sub-Themes (Intervention Group).

Impact on Child’s Nutrition
● Improved
“[The program has] had a huge impact on [my child]. [W]e were always … a free feed family …the fridge is open, the cupboards are open, take what you want, when you want. [My son is] nine so what he wanted was cookies, and sugars, and sweets, and starches all the time. [W]e do so much more meal planning now than we ever did. We sit … and figure out the whole week…. [On] ‘eat whatever you want Friday’ … he won’t eat a whole bag of cookies… which makes me happy…. [He says] ‘I ate my sub I’m going to eat an apple first and then I’ll have my cookie.’ … Whereas before he would’ve been like ‘can I have two? … three?’ Both [of] my… children, but definitely my son [program participant], has been [making] significantly better choices.”
“[T]wo weeks ago, we went to McDonald’s and [my child] made a comment going ‘Mommy I don’t really like McDonald’s anymore.’ … But he used to want McDonald’s all the time.”
“[My child’s nutrition has] changed a lot. She’s looking more at what’s healthy for her, compared to ‘I’m just hungry and bored and wanna eat.’”
● No Impact
“We try to eat… a lot of vegetables, and a lot of fruit, and…we’re already trying to do a lot of substitutions with a lot of vegetables, you know, tofu, beans, etc. … We’ve been doing that all along, so nothing’s changed for her.”
“I made it a goal even before the program… [to] feed [my child] better than I feed myself. … I made it a goal when she started eating sold food … to make sure that she was introduced to tons of vegetables and fruit, because it wasn’t the same for me growing up.”
Impact on Child’s PA
● Increased
“[My child’s behaviours] completely [changed since starting the program]. [H]e’s probably watching half as much TV now as he did three months ago… and … [he’s] wanting to go outside, wanting to play games, wanting to explore … anything [to be] outside more… And he was never that kid before.”
[My child] sees me moving more. And she wants to participate, so … I did a 100 squat challenge every day for 30 days… and she would join in with me. … it’s acceptable behaviour to her now, to move more and to exercise. [S]he’ll come to me… and [say] ‘it’s time for exercise.’”
“[Before the program my child] was signed up for nothing, and now she’s signed up for 3 things. … So she’s doing karate, yoga, and skating.”
● No Impact
“[My child] was super active before we started [the program] and she’s still really active today. … I think the only thing that I’ve changed is that.… I’m going to be enrolling her in some more programs for exercise and things like that.”
Improved Own Nutrition
“I’ve been… more purposeful to make extras for dinner because that’s one of the healthier meals that we eat [because] we eat as a family. … So I’ll make sure I’ll have enough leftovers for… myself for lunch, versus eating a small bowl of chips.”
“Grocery shopping has just changed. [I stick] to the outside perimeter of the grocery store versus going deep into the aisles. … [And when I’m] making muffins… I’ll just throw the carrots in. … I’m sure it’s not even a lot, but again, it’s changing the flavour, texture, getting the taste buds more used to having the sweetness of the carrot and the pineapple in the muffin instead of the sweetness of chocolate.”
“The program has definitely [had] a positive impact [on my dietary intake]. … I still struggle with stuff like eating enough fruits and vegetables. … My major goal when I was working with the coach was my sugar intake, that’s what we ended up focusing on. … I realized the other day that my coffee was too sweet and I reduced how much sugar was in it by one teaspoon and then it tasted so much better. I’m losing my taste for sugar for a little bit.”
Impact on Own PA
● Increased
“I think [what] the program did for me was [help me] understand how important my routine was and how much of an impact it was having on my life. So…the last four weeks I’m up early and working out, I’m making breakfast for my family before I get to work instead of rolling out and [saying] ‘we’re grabbing [fast food] today.’ [Before] I would be like ‘here’s a donut, here’s a cupcake. … And I think if I hadn’t gone through the program, I think I’d still be that way.”
“I’m doing more practical activity … like raking and shoveling and playing, skating, skipping, that kind of stuff. It’s not that I’m going to the gym, ‘cause I’ll never be a gym person, is what I have learned over the years. … But [the program] has just spurred me to try and be more active so, it’s … in the back of my mind all the time.”
“[Because my job involves a lot of sitting] I can’t incorporate a lot of walking, but I did start… parking further, in parking lots. …[When] sit[ting] in client meetings all day …started standing up, even between the meetings, and I started stretching a little bit more instead of just sitting at the desk and waiting for the next client to come.”
● Mental health
“[My coach and I] were talking about how when I’m feeling stuck, like literally just getting up and moving your body, can snap you out of a funk.”
Impact on Family’s Nutrition
● Improved
“We’re eating healthier because we are following our meal plan, we’re pre-planning our meals so we know what we have in the house and that we make sure we have enough for the rest of the week because I don’t want to go to the grocery store four-five times a week, now it might be twice.”
“I’ll just buy one of those vegetable trays from the grocery store and just put it out and [my children] eat it!”
“All the kids [love salads now]. One night… was a huge success, half [my son’s] plate was just salad, just naked veggies. … And before [the program], I don’t think that they would have been.”
● Changed food environment
“I think [my family] feel[s] like they’re getting a little bit more of a choice [of foods] now. Like if I call home and say we’re having this and this and this, [my children will] say like ‘oh can we have the green beans instead of that?’… [I say] ‘sure, we can switch those.’ …. [I]f I come home and I… need to then cook and then we eat, its 6:30–7:00. Whereas if [my family members] help, I get home, we eat, and then we can spend time together.”
“I now go through two bags of apples a week… and two bundles of bananas. … [Before], my bananas would just kind of rot, and then I’d make banana bread.”
“[We have been looking for] different [food] options. Like, the kids are even saying, ‘well, that’s not a good option, Mom. Let’s do this one.’ … So, having them buy into it is much easier. … And it’s just more of a conversation we have [about healthy eating], versus mom just plops stuff in front of them.”
Increase in Family’s PA
“We’ve been skating, we do skipping, and we were out raking yesterday. So [the program] pushes me…to try and do something everyday. Whereas before there were days where… I would come home and just be too tired and we wouldn’t do much at all.”
“[Physical activity is] easier because now …on days when I don’t feel like doing [physical activity], [my husband will] say … ‘okay, are we going to go on a walk with the dog tonight’ or ‘we’re going to take kids to the park, and on days where he doesn’t feel like doing it, then I’m the one pushing him. Whereas before it was both of us …watch[ing] TV.’”
“Friday nights [used to be] pizza and a movie nights. … And chips. …[V]ersus [now it is family] gym night. … Like, that’s a big change.”
Modeling Behaviours
“[The program is a] reminder that everything I do in my life, my children are watching me. So whatever I’m doing that I am a model for them. So if I’m modeling very lazy lethargic behaviour, watching TV all the time, not keeping active in my lifestyle, that’s what my kids are going to… think is okay and it’s going to trickle down because that’s what my parents were like. …[A]nd it trickled down into my life and I want to stop that now. I want my children to see what a healthy [life]style looks like.”
“I’m being accountable for what [my children are] putting in their bodies. … I knew what I was buying wasn’t the healthiest for them, but it was something that they like. So, I was [starting] to realize ‘okay, well just because it was something they like it doesn’t mean that they need to indulge in that.’”
Overall Program Experiences
● Increased motivation
“[The program] helped me to get the first step to being motivated to doing something … literally just moving.”
● Positive
“I loved the fact that you guys come to the house [for follow-ups]. [Because] … I’ve got a kid, so … getting out of the house is a whole production.”
“[The program] was just nice and… an overall reminder just to focus, slow down, look at what it is that we’re doing, and in my case, it was good to reinforce that … we’re doing the right things. …[Y]ou get so stuck in your routine … that you don’t slow down to actually look at what it is that you’re doing, and why, and is it working. So it was kind of nice to have a little bit of a review.”
● Future directions or improvements
“I thought it was a really great program… I guess maybe if I was to say anything it would be to like give two different options to people [for the education sessions]… of being flexible, versus having … deadlines and check points. My personality is that I will either try and … rush through it at the start or … do it at the very end, but if there’s deadlines I’m always committed to meeting my deadlines.”
“I think there are so many more people [who] could benefit from the program …. [T]he program is so impactful for me that I think… it should be offered to every kid in school. [I]f their parents don’t take it, they don’t take it, but this sort of stuff needs to be taught more in our school at a younger age.”
“The only recommendation was to do the webinars… an audio version of them.”

**Table 10:** Corroborative Quotations for Post-Intervention Themes and Sub-Themes (Control Group).

Increased Awareness
“[My child and I] talked about healthy [eating], like she always knew what a healthy versus unhealthy food was, but like when [she’s] five and there’s a bag of chips and apple I mean obviously she is going to choose that. So I find now that she is more likely to go and choose one of her healthy snacks without me having to remind her.”
Impact on Child’s Nutrition
● Improved
“We are hard on fast food now. …instead of just eating fries or cheese pizza [at the mall] she would go and get white rice, broccoli, or like shrimp. …[E]ven though some of that stuff’s not…made the best, at least I know … that stuff is going to fill her up [better] than the other stuff.”
“Even getting [my child] things like a protein bar right, because at least that’s better than eating a chocolate bar. … So, we would buy something that was a little… healthier on the go, because he’s usually running out the door in the mornings. … And, [I am] buying the … cut fruits and vegetables so that it is already ready for him to grab….”
“[My child has] always been a pretty good fruit eater. But we tried to introduce new foods, expand the repertoire… some vegetables, some protein. … [He is] eating carrots now, celery… we tried some beans, black beans and kidney beans. … Scrambled eggs.”
● No Impact
“I don’t think [nutrition] necessarily changed for [my child]. It wasn’t necessarily the dietary stuff, for her. … There’s never been a lack of healthy options in the home.”
Impact on Child’s PA
● Increased
“[Before the program my children would] come home… get a freezie or … a pack of gummies, and they would just sit. … Most of the time my oldest would always fall asleep then she would complain of headaches, so this summer… she wasn’t the one steady participating [in this program] per se but she picked up off of the younger one [who was participating]. [T]he younger one … now …[has] a routine, it’s normal for her. So now if she’s watching TV too long, she gets fidgety. … It’s almost like her body’s telling her like you need to get up and do something … She’s like ‘okay, I’m going to go play’ … [the older child is] getting better, too.”
“We definitely are more active now. … Like [my child] snowboards now, he plays hockey, so I volunteer on their hockey team. …. Before [the program] I’d put him in maybe one sport, but now I’m … trying to get him to do more. … So I’ll be taking him more often because it’s really important… it’s fun and we can be active and spend time together.”
● Enjoyed using pedometer
“I do think [my child] tried to get more steps in with that pedometer. She’d be sitting still and then stand up and walk around and then look at the pedometer and stand up and look at the [step count]. And I would remind her, ‘It’s supposed to be honest, so when you’re walking… you don’t need to do extra steps.’ But, I suppose it is nice that she did get excited by it, and I couldn’t squash that, it was great.”
“[My child] was very conscious of [step counting], and of course he liked [it] because he got more steps the days he played soccer… he was into it.”
● No impact
“I don’t think [my child’s PA was] really impacted that much. I think… out of the two of us, [my child is] far healthier than I am. Probably because I realize how unhealthy I can be, and want to make sure I’m not instilling those bad habits into her. So I think for her, it was … repetitive information. … She listened to what I had to say, she watched a few videos with me, whenever it was directed towards the child portion, and then just went on her way. I don’t think it really made a significant difference… she’s always active.”
Impact on Own Nutrition
● Improved
“[Now] I [have] been like ‘nope, I don’t need to get those chips.’”
“When I have one carrot, or two, you know, just leftover vegetables… I just puree them…freeze it, and then I use it in my pasta sauce.”
“[I]ts not that I don’t like vegetables, it’s that… I find them like boring, and so … I try to make [healthy food] fun for me, too. But… [also trying to stay] away from … bad fats and everything like that.”
“[The program helped with me] knowing if I do want a snack, I don’t need the whole bag… [of] chips …. [N]ow, I put some in … a little kid bowl. … [I]f I eat a bag of chips with 1100 calories, that’s more than half of what I should be eating in a day… and I’m still hungry,’ so that was like a big ‘what did you just put in your body’ [realization].”
● No Impact
“I wouldn’t say that my eating habits improved all that much but definitely I’m more aware of it.”
Impact on Own PA
● Increased
“I play baseball during the summer, …I signed up for yoga, which I’ve always been intimidated by. … [I also have been] going to [the gym at work] after work, and now… I park further away so I walk 15 min to work, and 15 min from work.”
“I started a new job in the Fall. … And I’ve got my office on the sixth floor and I have stuff to do on the ninth floor and first floor and second floor; so I’m taking the stairs as much as I can.”
“Because my son is more active, I’m more active, I don’t just sit around all day. I’m …going out and doing sports with him …before I would probably never. I mean I would have but like it’s a drag. … I still find it exhausting, but I much rather go out and do things with him if it makes him happy.”
● No impact
“When I did the first surveys and read the modules, I had a plan, well not exactly a plan, a thought that I would increase my walking, kind of throughout my work day, like take regular walk breaks. … But it just, it hasn’t happened.”
“I can’t seem to find time in the days … the days are just so busy, it’s hard to kind of find a time. I mentioned it to a colleague also, who said she would be interested in going walking… but we have never found a time that we’re both free to do that…which is depressing.”
Impact on Family’s PA
● Increased
“I think [we have] increased physical activity… heading down to the park after school or going for a walk, [because] … it’s really easy sometimes when you’ve had a really long day…to say ‘No we’re just going to stay in.’ But if [my child is] asking to go on a walk, nine out of ten times we’re going to say yes. … We’ll take her to the park, or go for a rollerblade, or whatever it is she wants to do. … So just being more active as a family… more frequently.”
“Before [the program] we [would] go for walks but it was like just around the corner and then come back with the dogs and that’s it. But right now, we spend more time together, because we don’t go anymore to the corner, we… go for example to [the park] and we walk the whole [trail]. … We walk, and walk, and walk, and we play together, and we run, we go to parks so, it’s keeping up [PA] together, and my family is helping a lot.”
● No impact
“To be fairly honest, I don’t think [the program has] impacted us at all. Probably because I went through it… at the beginning. We were all reading through it, and paying attention to it, and being cognizant of it, and then we just kind of got busy and fell off the track, right? So, [my child]… was excited to watch the videos with me, and we discussed it, and once that piece was over, it was kind of just over. There was nothing, there was nothing more to kind of, pull through.”
“I would say [the program had no impact on my family’s PA]… my two sons are very active, my daughter doesn’t really do extra-curriculars yet because she’s small, but she’s kind of active throughout her day.”
Impact on Family’s Nutrition
● Improved
“We’ve been eating less canned food, too. … [Because] there’s a lot of salt in there.”
“Before going back to school, I would hit Costco and just buy boxes of things … and there’s … a drawer [in our home] so they put their hand in and grab whatever they want for a snack… but it’s all packages. … gummies, granola bars … Jello …juice boxes. And now they they get water bottles, so they have nice water bottles… One’s got a Caesar wrap in her lunch for tomorrow and the other one has a cheese quesadilla on …whole wheat naan … instead of … before it’d be morning of, I’d just be grabbing handfuls and throwing it in lunch… grabbing a packet of ‘garbage.’”
● No impact
“Once I was through all the videos and stuff I kind of fell off [the program]. So, overall the impact was more just me, and being cognizant of my, I guess bad behaviours, is essentially what it is, my bad eating behaviours. But overall, as a family, it hasn’t changed.”
“My husband [went to Costco]… and he said, ‘well, I’ll just buy some food for us,’ [because] I was out, and so they brought a whole… prepared lasagne. [Because] it was only 13 dollars, and it was really good, you know? … So, I’m asking, you know, ‘Where’s the box, [what was the nutritional content] in it?’ … They don’t care. … [If they could, they would eat] that lasagne thing everyday, and the apple pie.”
Modeling Behaviours
“My husband… is now down 43 pounds. … He doesn’t drink sugar anymore… the fridge mostly [has] water… he’s coaching… football… and out he’s out in the heat right now, where[as] before he would come home, get on his laptop, watch TV, and then have his phone. … So, now three nights a week he’s out coaching football. … It blows my mind because he doesn’t feel sluggish anymore. … sugar… was … the biggest thing for our family. [O]nce we went through that massive first two weeks of … shock to your system, where everyone felt horrible… then we started feeling better….’”
“[My child] joined track this year, she’s joined the volleyball team so I think [me changing my habits] helped to motivate even her to be… more active as well. …. [S]he sees me being more active instead of… coming home and just slumping on the couch, which isn’t good. …. I was coming home so exhausted… so she just noticed… that I’m not looking and feeling so exhausted.”
“Obviously I don’t wanna see my husband eating a hamburger, and I would be eating vegetables. … So, I wouldn’t like that. … My husband doesn’t like vegetables, but he’s eating them… because he wants to help me and help my son too, because obviously my son would say ‘okay why [is] daddy not eating that and why I am eating this?’ … So, all of them are changing.”

**Table 11:** Corroborative Quotations for 6-Month Follow-Up Themes and Sub-Themes (Intervention Group).

Overall Impact of Coaching
● Positive
“It was an interesting experience having a life coach, as it made you realize how much all the aspects of your life are correlated. So that made a big difference.”
“I loved the start of [the program] I don’t think I would’ve done as well if I hadn’t had someone to talk to. Personally I probably would have loved [coaching] to go on a lot longer. But I think that’s why people who join programs are successful, it’s because they have someone they are accountable to for a longer period of time. … I felt like I could talk to somebody about what my struggles were, who was going to not just listen but help me overcome some of them.”
● Improved mindset
“I think [coaching] was probably the best part [of the program]. I think just working on that mental stuff really helps translate into everyday life. … Like, after the sessions, you feel better. And so then you’re more inclined to … eat something healthy and go for a walk as opposed to come home and sit and eat chips on the couch, you know? … More motivated.”
“[My coach] had me actually walk away from a fitness company that wasn’t serving me… the relationships I had built with this fitness company were harming my mental health more than helping my physical health. So, she had me walk away from that, which, for mental health was really good … [I began] at-home workouts …which, with my busy schedule, worked really well for being able to fit that in at home.”
“My friends can see the struggle of ‘I don’t want to eat that’ or ‘I want to lose weight,’ but [my coach] was able to take me to a deeper level of why I was so enamoured with food … and you know … the other things I needed to deal with and heal to stop replacing them with food. … So, [coaching] was probably the best part of that entire thing.”
● Change in perspective
“[My coach and I] talked a lot about happiness and what does that weight really mean and the end of it… [I realized] ‘you know what, I’m just as happy whether I’m 5 pounds up or whether I’m 10 pounds down.’ … I don’t stress [about weight] nearly as much as about it as I did before.”
“It’s hard to keep [behaviour change] going and that was something that my life coach sort of helped drill into me, that just because you’re not doing it exactly the way you want to do it now doesn’t mean you should stop, right? So, I think that’s something that I keep in my head, you know, just because you haven’t gone to the gym in 2 weeks doesn’t mean that now you can never go again. … you can start again.”
“[My coach] allowed me to see that I can get healthier, but still love who I am. It doesn’t mean I don’t love myself or the size I’m at, or the person I am, currently.”
● Long-term strategies for behaviour change
“I still will sometimes force myself to go back and look at things the way [my coach] would have made me look at it. … I’ve gone back and read my notes about things that we’ve talked about… she has changed my thinking on so many important things [which] has really helped …going forward. And then that does change things like the physical aspect and the nutrition, because I’m looking at it from a different lens.”
“The main thing that the life coach kind of instilled on me [was] that you have to kind of put yourself first because if you don’t then everyone else kind of suffers too, so it’s the biggest thing that I took away from it.”
“[My coach] encouraged me to do some journaling… and I’ve continued to do that. And then she just gave me some just tips on how to, how to get that motivation going again so when it starts to get to a low period, what to do to kind of kick start that.”
Impact on Own and Family’s Nutrition
● Meal preparation
“[Meal preparation] saved me a lot of time and energy just making things simpler, knowing my family’s eating better. I’m not running to the grocery store every other day trying to grab stuff to eat. So, [the program] just improved our life in a lot of ways.”
“We go grocery shopping on Saturday and buy everything we need for the week, so there’s no stress that way. … And it is something that I’ll just continue, because it works well for our family. … We know exactly what’s there, and some of the things I will prep on the weekends, and so it’s easier to then cook on the weekday.”
“We were a big granola bar family or cookies or chips, the things that are really super high in carbohydrates, and quick to grab. But if we do meal prep, then we’re a lot faster to grab like a salad or some vegetables that are already cut, or fruit.”
Barriers
● Stress
“Probably more than 6 months ago I was going to the gym more often, and making a better effort with my diet, and making sure my daughter was getting more activity as well. And now it just, because of time constraints, and probably a bit of stress, and probably a bit of anxiety, it’s sort of slid back a bit. … Eating has been a challenge and we’ve definitely been relying a little more on restaurants than we want to.”
“I still notice when I’m stressed, I do eat a lot and then it doesn’t help and then when I get sick … I use that as an excuse to like not do anything. … It’s … that habit-forming thing, you have to do it for um at least 21 days before it sticks. And so, I have to just get back in to that and stick with it.”
● Motivation
“As I’m watching the leaves start to change I can feel … excuses starting, like, ‘Oh, well I could put something in my lunch time so I don’t have to go for that walk.’ …[T]hose excuses … won’t better myself, but it’s a pattern I’ve always done and it’s very normal for myself. But I’m trying to go against those and work against what my natural inclination to continue those walks.”
● Time
“One of the biggest challenges is now [my children] have something every single evening, and we are literally flying from where I pick them up at their after-school program, home, eat dinner, and then out to something for them. They continue to stay active. … But that forces me to go and be sedentary most of the time, at their things, watching them be part of those fun activities.”

**Table 12:** Corroborative Quotations for 6-Month Follow-Up Themes and Sub-Themes (Control Group).

Prevention
● Habits in child
“[I am] trying to develop health habits long-term so that when [my child is] a teenager, [and] he has more access to food, that he makes good choices.”
“I think [I am] realizing that my decisions, and [the] behaviour [I] model for [my child] could… become a struggle, and I don’t want that for her. … Now [I’m] realizing that my decisions aren’t only decisions for me. And I think that’s been probably the biggest part [that helped with behaviour change], is realizing I don’t want her to ever [struggle with health].”
● Developing adverse health effects
“Some of the questions [in the questionnaires] about your like physical things like ‘has this stopped you from doing this?’ I never thought at my age [that I would] answer yes to those things but like I was literally [having] a hard time getting off the floor.”
“I am getting older and my health may deteriorate outside of my control, so the things I can control I feel like I should control like keep my weight down, keep my heart active.”
Changed Perspective
● Toward behaviour change
“I don’t have any resounding health issues. So, I feel like that’s almost been an excuse. ‘Well, it’s not that bad. I don’t have high cholesterol, I don’t have diabetes, I don’t have thyroid issues.’ And I think I needed to get past that mind frame of thinking it’s not that bad, and being like, ‘You know what? I’m not happy with what I see in the mirror, and I need to change that. And the only person that can change that is me.’”
“I do have to give myself a little bit of credit. I am doing a much better job [of being healthier] than I have previously. … This year my goal [was] living a healthier lifestyle. So I didn’t choose the, ‘I’m gonna join a gym and lose weight,’ I chose the overall healthier lifestyle.”
● Prioritized themselves
“I try to make sure I carve out ‘me time’ in the day [now], so if they choose to join, that’s awesome.”
“Cardio helps me a lot, because I make that my time. … I’ll listen to a podcast, or I’ll watch YouTube. … Zone everyone else out. … [Which helps with my mental health]… It helps me sleep better, too, which is great.”
● Improved mindset
“I think that it [was] maybe not necessarily the nutritional information and stuff I was watching, but kind of the subliminal messages of, ‘You don’t need to be afraid of [changing behaviours].’ And, people aren’t viewing you and judging you the way you are judging yourself. So I think that has kind of been my biggest takeaway from the whole process.”
“Exercise makes me relax. … Before I [was] always fighting with everybody and I was so stressed. … And now, I started going to the gym and whenever I’m stressed I just go and run … and I come [home] like new. So I think that’s what helps me [continue with behaviour change].”
Barriers
● Motivation
“Before I had like more time to focus on [health behaviours], and that was my focus and like being somebody that has grown up with weight issues it always has to be a focus. … Like I’m not going to wake up one day and… be like I really feel like a kale salad right now. … So it has to really be a conscious decision for me… to eat in a healthy mindful way. … And I just haven’t been in that mind set right now.”
“[It has been] just like super busy time at [work] right now. Like I didn’t even stop today for lunch. … It [has] just been crazy. … [But] hard it’s that like cognitive dissonance like I know I should be [eating better and being physically active] and then I’m not doing it, and then I feel guilty because I know I should be doing it, and I’m not doing it.”
